# Integrative Transcriptomic Profiling of the Wilms Tumor

**DOI:** 10.3390/cancers15153846

**Published:** 2023-07-28

**Authors:** Simona Lucija Avčin, Klementina Črepinšek, Barbara Jenko Bizjan, Robert Šket, Jernej Kovač, Blaž Vrhovšek, Jerca Blazina, Olga Blatnik, Robert Kordič, Lidija Kitanovski, Janez Jazbec, Maruša Debeljak, Tine Tesovnik

**Affiliations:** 1 Department of Haematology and Oncology, University Children’s Hospital, University Medical Centre Ljubljana (UMC), 1000 Ljubljana, Slovenia; simona.avcin@kclj.si (S.L.A.);; 2 Faculty of Medicine, University of Ljubljana, 1000 Ljubljana, Slovenia; 3 Institute of Special Laboratory Diagnostic, University Children’s Hospital, University Medical Centre Ljubljana (UMC), 1000 Ljubljana, Slovenia; 4 Department of Pathology, Institute of Oncology Ljubljana, 1000 Ljubljana, Slovenia; 5 Department of Pediatric Surgery, University Children’s Hospital, University Medical Centre Ljubljana (UMC), 1000 Ljubljana, Slovenia

**Keywords:** Wilms tumor, NGS, miRNA profile, mRNA profile, universal biomarkers

## Abstract

**Simple Summary:**

The Wilms tumor is the most common kidney cancer in children. This study characterized complete miRNA and mRNA profiles using a comprehensive NGS sequencing approach to identify miRNAs as transcriptome regulators involved in the Wilms tumor. By analyzing two independent groups of kidney samples with matched controls, we identified 43 miRNAs that are independently differentially expressed in the Wilms tumor irrespective of tumor histological type. Our results provide new insights into the role of transcriptomics in the Wilms tumor and identify universal miRNAs as potential biomarkers for early detection and targets for prevention and treatment of the Wilms tumor.

**Abstract:**

Our study aimed to identify relevant transcriptomic biomarkers for the Wilms tumor, the most common pediatric kidney cancer, independent of the histological type and stage. Using next-generation sequencing, we analyzed the miRNA profiles of 74 kidney samples, which were divided into two independent groups: fresh frozen tissue and formalin-fixed paraffin-embedded tissue samples. Subsequent mRNA expression profiling and pathway analysis were performed to establish the interplay and potential involvement of miRNAs and mRNA in the Wilms tumor. Comparative analysis, irrespective of post-dissection tissue processing, revealed 41 differentially expressed miRNAs, with 27 miRNAs having decreased expression and 14 miRNAs having increased expression in the Wilms tumor tissue compared to healthy kidney tissue. Among global mRNA transcriptomic profile differences, cross-sectional analysis suggested a limited list of genes potentially regulated by differentially expressed miRNAs in the Wilms tumor. This study identified the comprehensive miRNA and mRNA profile of the Wilms tumor using next-generation sequencing and bioinformatics approach, providing better insights into the pathogenesis of the Wilms tumor. The identified Wilms tumor miRNAs have potential as biomarkers for the diagnosis and treatment of the Wilms tumor, regardless of histological subtype and disease stage.

## 1. Introduction

Renal tumors are among the most common solid neoplasms in childhood, representing 6–7% of all pediatric malignancies and affecting 1 in 10 thousand children. Due to their embryonic origin, they presumably develop into nephrogenic rests (NRs) with morphological and molecular analogies to kidney development [1]. In up to 15% of nephroblastoma cases, commonly known as the Wilms tumor (WT), malignant development is associated with germline mutations in cancer risk genes, such as single nucleotide variants or deletion of the WT1 gene, and some cancer predisposition syndromes [2]. The main causes of WT are somatic mutations in *WT1*, *CTNNB1*, *AMER1*, *TP53* and *MYCN*, genes associated with epigenetic chromatin remodeling [2], microRNA (miRNA) processing genes (*DROSHA*, *DICER1*, *DGCR8*, *XPO5*, and *TARBP2*) [1,2] and loss of heterozygosity at 11p15 [3].

The differences between the formation, expression and cell biology of miRNAs in normal and tumor cells are the subject of extensive scientific research on many cancer types [4,5]. miRNAs are short non-coding RNA molecules, 19–25 nucleotides in length, that are involved in the regulation of gene expression [4]. The binding of miRNAs to the target regulatory regions of mRNA, as the most studied regulatory 3′end of the untranslated region (3′UTR), affects the stability and translation of mRNA. It is estimated that miRNA molecules regulate approximately 50% of all mammalian protein-coding genes, where individual miRNA species can bind and regulate multiple mRNAs [6]. Tissue-specific miRNAs are involved in the formation, growth and differentiation of cellular structures and tissues [7,8] and can be involved in tumor pathogenesis. Altered miRNA formation and expression have been reported in several pediatric solid tumors [1,9]. According to the results of recent studies, alterations in miRNA expression could play a key role in the formation of the WT, where malignant transformations could be a direct result of disturbances in organogenesis and growth of embryonic tissue [10]. Although the treatment of the WT is successful, advanced comprehensive approaches are needed for personalized therapy. The main goal is to improve outcomes by combining the results of recent research, including molecular genetics, with potential novel biomarkers independent of WT histology and prognostic significance [5,11]. Thus, our study aimed to identify WT-related transcriptomic markers independent of the WT subtype and tumor stage, which represent potential diagnostic and therapeutic targets.

## 2. Materials and Methods

Our aim was to identify differentially expressed miRNAs in the WT compared to samples from healthy kidneys (HK). In the second part, we confirmed these results by broadening the study to include the examination and comparison of WT and HK formaldehyde-fixed paraffin-embedded tissue samples (FFPE). In the third part, we specified the impact of differentially expressed miRNAs on mRNA expression in nephroblastoma tissues. We used in silico analysis to predict the correlative effect of miRNAs on mRNA expression and evaluated the results by analyzing the mRNA expression in renal tissue.

### 2.1. Patient Inclusion Criteria

The included patients were treated for the WT at the Department of Hematology and Oncology, University Children’s Hospital, University Medical Centre Ljubljana, in the period 2000–2019. Age at diagnosis, sex and WT subtype were assessed. The study protocol was approved by the Republican Medical Ethics Committee (Consent No. 0120-3750/2015-2) after submission of the protocol, ensuring that the study would not lead to additional interventions or increased patient burden, considering all the principles of the Declaration of Helsinki. The WT with its stage and risk assessment was determined according to the SIOP RTSG pathology protocol after surgical excision of the tumor with subsequent central pathological examination. All patients, their parents or legal guardians provided written informed consent prior to enrollment.

### 2.2. Whole-Exome Sequencing Analysis for Exclusion of Germline Variants Associated with the WT

Non-syndromic patients without germline mutations in the WT-related genes were included in the fresh frozen kidney tissue (FFT) sample group. Whole-exome sequencing (WES) was performed to exclude possible germline genetic variants associated with the WT. Blood samples (K-EDTA extraction) were collected for DNA isolation using the FlexiGene DNA Kit (Qiagen, Hilden, Germany). After the preparation of next-generation sequencing (NGS) libraries (Illumina^®^ DNA Prep with Enrichment, Illumina; IDT xGen Exome Hyb Panel v2, IDT), WES was performed using a NovaSeq 6000 sequencer (Illumina, San Diego, CA, USA) with a minimum 100-fold horizontal coverage of the analyzed regions.

### 2.3. miRNA Expression in Wilms Tumor Fresh Frozen Kidney Tissue

FFT of WT and HK samples were obtained after surgical removal of the affected kidney following preoperative chemotherapy. The tissue samples were evaluated, resected, microscopically examined according to SIOP pathological guidelines and cryopreserved in a deep freezer at −80 °C until further processing. FFT samples were cryogenically homogenized, and RNA was isolated using the miRNAeasy isolation kit (Qiagen). The quality of the isolated samples was assessed using the RNA Pico 6000 reagent (Agilent, Santa Clara, CA, USA) before further processing. Small RNA libraries for NGS were prepared from isolated RNA using the NEBNext Small RNA Library Prep Kit for Illumina (New England Biolabs, Ipswich, MA, USA) and sequenced at more than 20 million reads per sample.

### 2.4. miRNA Expression in Wilms Tumor Formaldehyde-Fixed Paraffin-Embedded Tissue

The miRNA expression results of FFT samples were confirmed with 54 FFPE samples from 27 WT patients (27 FFPE WT samples and 27 matched FFPE HK tissue samples). FFPE slices were cryopreserved at −80 °C until RNA isolation. For every FFPE sample, RNA was isolated from five slices using the RNeasy FFPE Kit (Qiagen). The quality of the isolated samples was assessed RNA Pico 6000 (Agilent). miRNA NGS libraries were then prepared using the NEBNext Small RNA Library Prep Set reagent kit for Illumina (New England Biolabs) and sequenced using a NovaSeq 6000 (Illumina). All FFPE miRNA NGS libraries were sequenced using more than 10 million sequences.

### 2.5. mRNA in Wilms Tumor Fresh Frozen Kidney Tissue

The mRNA expression in WT was determined on RNA isolated from FFT samples of WT and HK tissue, which were used for miRNA determination. Samples for mRNA expression were prepared using the NEBNext Globin&rRNA Depletion Kit Human/Mouse/Rat (New England Biolabs) and NEBNext Ultra II Directional RNA Library Prep Kit for Illumina (New England Biolabs). mRNA expression profiles were determined after mRNA sequencing with 100 million reads per sample using a NovaSeq 6000 system (Illumina).

### 2.6. Bioinformatic Analysis

To define germline genetic changes, WES data were analyzed using the bcbio_nextgen software toolkit (version 1.2.9) [12]. The analysis included sequence alignment on the human genome reference (hg38) using the bwa tool [13] and identification of genetic variants using the GATK4 genetic alteration detection software package. Genetic variants were annotated and filtered using the VarAft software package [14].

miRNA profiles in FFT and FFPE WT tissue samples were determined using the sRNAtoolbox web server bioinformatics toolkit [15]. The analytical pipeline of the bioinformatics toolkit includes the removal of adapter sequences, alignment of reads to the human genome (GRCh38_p13) and annotation of analyzed sequences based on the miRBase database (miRBase: release 22.1, sequences: Homo sapiens). To determine the differentially expressed miRNAs between the compared groups of WT and HK tissue samples, the bioinformatics tool sRNAde was used with five comparison tools (DESeq, DESeq2, EdgeR, NOISeq and *t*-test). As significantly differentially expressed miRNAs, we identified those miRNAs that had more than one identified read and had false discovery rate (FDR) values less than 0.05, as determined by at least three comparison tools.

Additionally, we aimed to identify High-Risk (HR) WT associated miRNA. To determine the HR WT miRNAs, we compared the expression of High-Risk FFT and FFPE samples with non-High-Risk (nHR) samples and HK. The characteristic WT High-Risk miRNAs were determined by a cross-sectional analysis of the two groups following the principles of the bioinformatics procedures described above.

The physiological significance of differentially expressed miRNAs was determined by in silico bioinformatic analysis with visual analytics of the centric network of statistically significantly expressed miRNAs (|log2FoldChange| > 2, FDR < 0.05) and the database of experimentally validated potential miRNA-target correlation miRTarBase, using the miRnet platform [16].

The differential expression of mRNA in WT was determined by bioinformatic analysis of mRNA NGS data based on the SnakePipes tool [17]. The Cutadapt tool [18] was used to remove adapter sequences and low-quality sequences. The pseudo-alignment algorithm Salmon tool [19] was used to quantify and count mRNA sequences. The R-library DESeq2 [20] was applied to display the basic differential expression metrics.

## 3. Results

### 3.1. Patients and Epidemiological Data

Between 2000 and 2019, 43 children with the WT were treated at the Department of Hematology and Oncology, University Children’s Hospital, University Medical Centre, Ljubljana. Among these, 37 patients had available FFT or FFPE samples of WT and HK tissues that fulfilled the inclusion criteria. Therefore, 18 boys (median age at diagnosis 3.17 ± 2.65 years) and 19 girls (median age at diagnosis 3.28 ± 2.70 years) were included in our analysis, all of Caucasian descent. All the patients were treated according to the SIOP RTSG protocol. No pathological or likely pathological germline genetic variants in genes associated with WT (HPO Nephroblastoma HP:0002667) were identified in the subjects.

Thirty-six patients (97%) received preoperative chemotherapy, and one child underwent upfront surgery. According to the anatomical stage classification, 18 children (48%) had clinical stage I, six (16%) had stage II, three (8%) had stage III, seven (19%) had stage IV and three (8%) had stage V WT, of which 8 patients were classified as HR WT (stage IV and V) and the other 29 patients as nHR WT (stages I–III). The median age at the time of nephrectomy was 3.17 ± 2.67 years.

### 3.2. Whole-Exome Sequencing Analysis for Exclusion of Germline Variants Associated with the Wilms Tumor

We identified no clinical signs, symptoms and familial history of any of the congenital syndromes associated with an increased likelihood of the WT. WES analysis performed in all FFT individuals on peripheral blood samples was negative for WT germline variants of WT-associated hereditary syndromes. FFPE cohort individuals were not analyzed for germline WT associated variants due to a lack of availability of DNA samples.

### 3.3. Differentially Expressed miRNA in Fresh Frozen Tissue WT Samples

WES NGS was used to analyze miRNA expression profiles in WT and HK tissue of surgically removed kidneys of 10 patients diagnosed with the WT. The histological characteristics of the samples included in the FFT analysis are shown in Supplementary Table S1. Due to a higher proportion of necrosis in one of the tumor samples and lower RIN value, which resulted in an unsuccessful small-RNA library, the necrotic sample (Sample 6) was excluded from the analysis.

**Figure 1:**
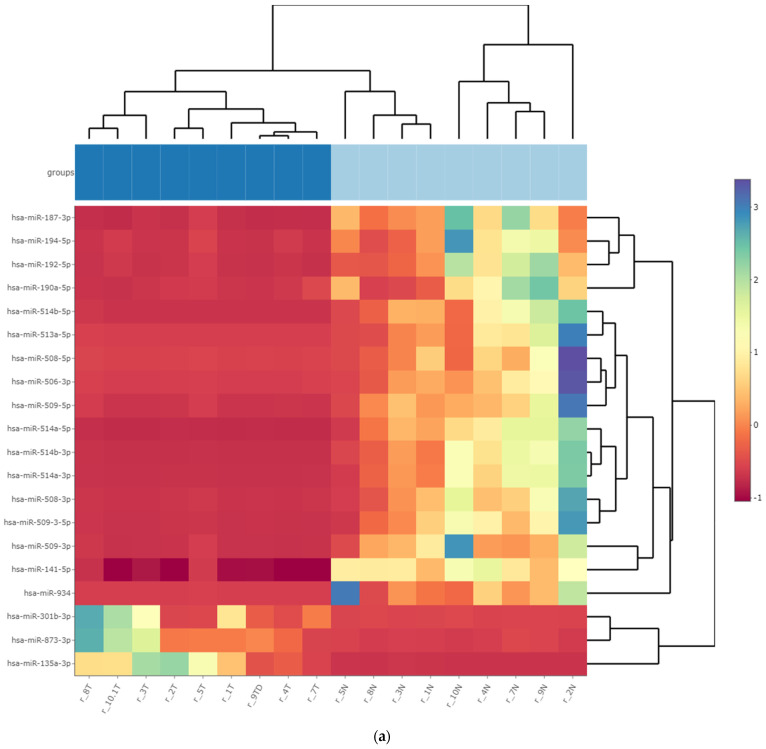

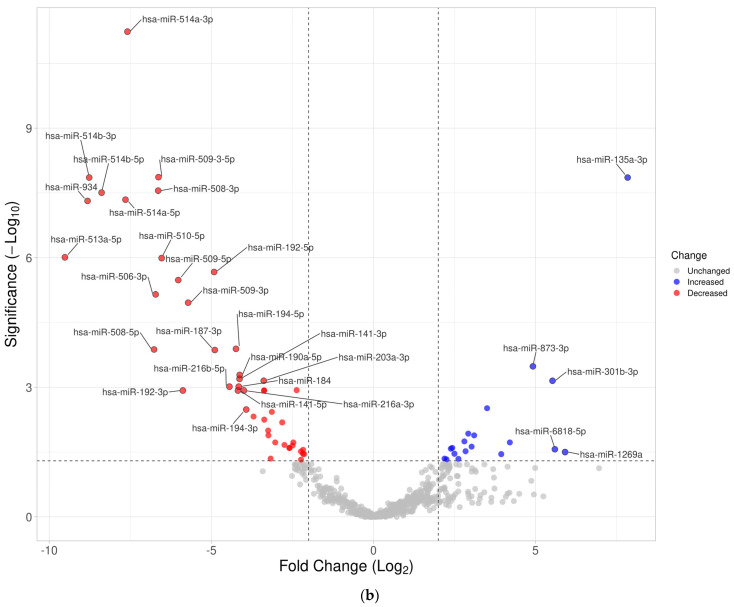
WT FFT miRNA differential expression. DESeq2 results of comparative analysis of significantly differentially expressed miRNAs in FFT tissue samples from tumor and healthy kidney (N = 9). A graphical representation of the results of the analysis is given in the form of (**a**) heat plot (WT samples in the groups section are indicated by dark blue squares, HK by light blue squares) and (**b**) volcano plot.

Comparative analysis of miRNA expression in the nine investigated patients, with matched analyzed WT tissue samples and HK tissue, revealed a variable number of differentially expressed miRNAs according to different comparison tools (Figure 1). Of these, 88 miRNAs had significantly reduced expression, and 39 miRNAs had increased expression in WT tissue, as determined by at least three comparative sRNA algorithms (|log2FoldChange| > 1, FDR < 0.05) (Supplementary Table S2a–c). The statistical significance of differential miRNA expression varied among the five different comparison tools which included different data normalization procedures.

### 3.4. Differentially Expressed miRNA in Formalin-Fixed Paraffin-Embedded Wilms Tumor Tissue Samples

**Figure 2:**
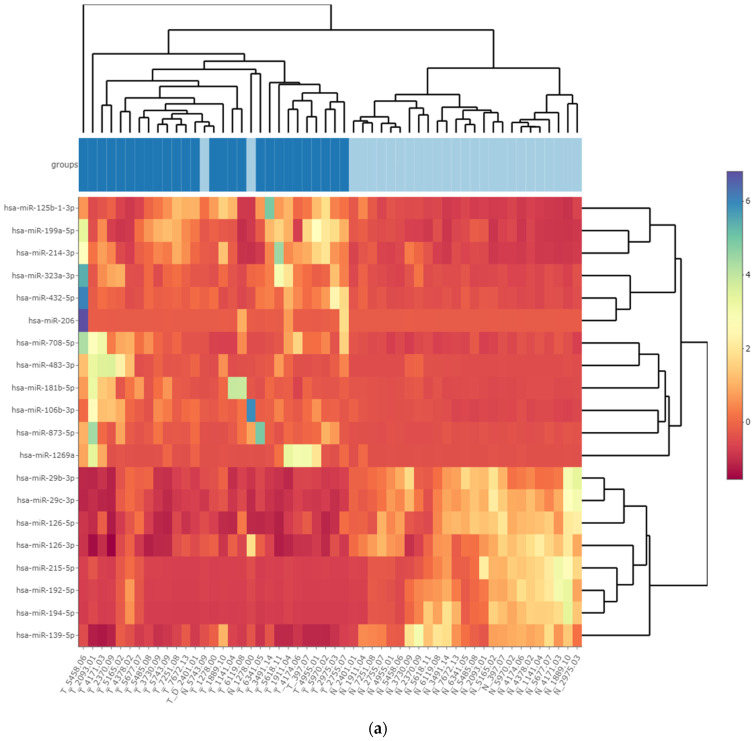

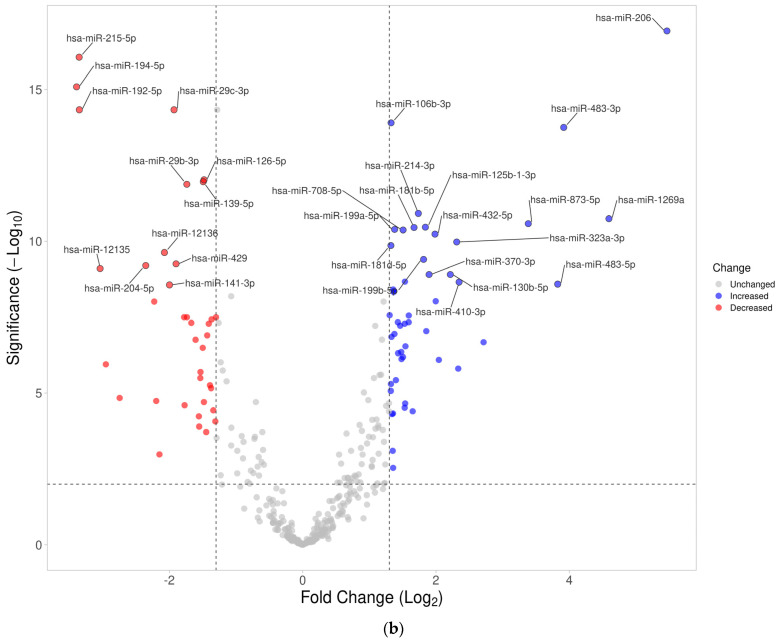
WT FFPE miRNA differential expression. Results of DESeq2 comparative analysis of significantly expressed miRNAs in FFPE samples of WT and HK tissue presented as (**a**) heat plot (WT samples in the groups section are indicated by dark blue squares, HK by light blue squares) and (**b**) volcano plot (N = 37).

The results of specific miRNA expression in WT tissue were confirmed by additional analysis of miRNA expression in 54 FFPE samples from a group of 27 subjects diagnosed with WT (Supplementary Table S3), which were not included in the FFT cohort. In this part of the study, miRNA expression in WT and HK samples was determined by NGS. The results of differential expression analysis for miRNAs varied between the different comparison tools (Figure 2). Overall, we identified 77 miRNAs with significantly increased expression and 51 miRNAs with decreased expression in WT tissues, as determined by at least three comparative algorithms included in the sRNAde toolbox (|log2FoldChange| > 1, FDR < 0.05) (Supplementary Table S4a–c).

### 3.5. Differentially Expressed miRNAs in the Wilms Tumor Irrespective of Tumor Histological Type and Stage

A comparative analysis of significantly expressed miRNAs in FFT WT samples and FFPE WT samples confirmed the decreased expression of 27 miRNAs and increased expression of 14 miRNAs in WT tissue (Table 1). To address variability in gene expression across different sample types (WT and HK), the miRNA counts were normalized using five different comparison tools, which consolidated differential expression miRNA profile results and decreased false positive results.

### 3.6. Differentially Expressed miRNAs in the High-Risk Wilms Tumor

In addition to characterizing the WT miRNAs irrespective of tumor histological type and stage approach, we also characterized WT samples for HR WT miRNAs. To address this, we compared miRNA expression in HR WT FFT samples with HK and nHR WT samples. The same was performed with the FFPE samples, and HR WT miRNAs were identified only if miRNAs were significantly differentially expressed in both FFT and FFPE High-Risk WT samples but not in non-High-Risk WT samples (|log2FoldChange| > 1, FDR < 0.05). Cross-sectional analysis of FFT and FFPE differential expression did not reveal any significant miRNAs for HR when HR samples were compared to nHR. In contrast, by comparing HR WT and nHR WT with HK samples, we identified 5 miRNAs with decreased expression and 6 miRNAs with increased expression in HR WT. Among these miRNAs, hsa-miR-363-3p had increased and hsa-miR-708-3p decreased expression in HR and not in nHR when sampled were compared with healthy samples (Supplementary Tables S5a,b and S6; Supplementary Figure S1a,b).

### 3.7. Cellular Processes Regulated by Differentially Expressed miRNA in the Wilms Tumor

**Figure 3:**
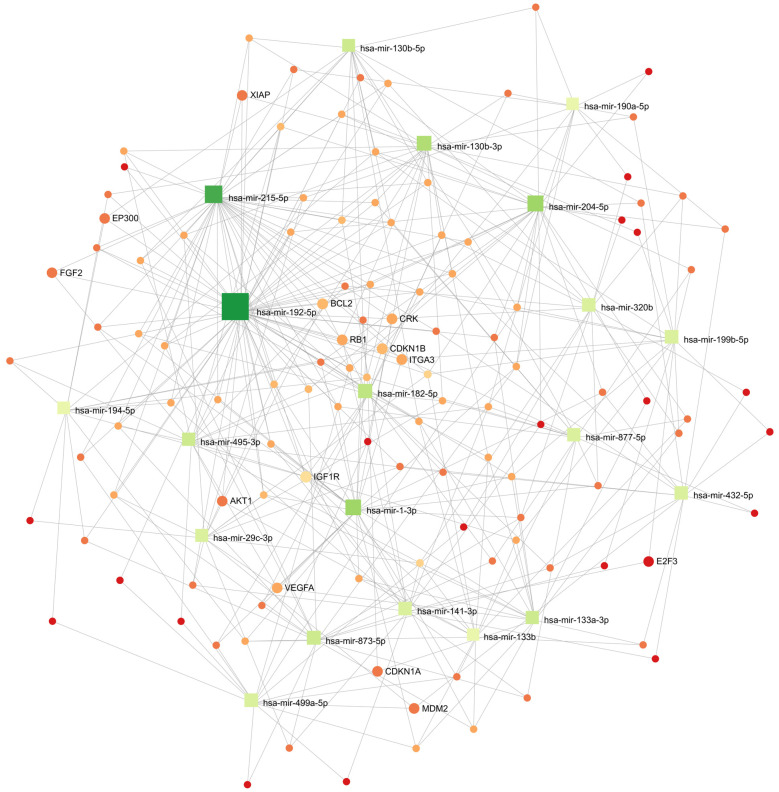
WT miRNA minimal network. Graphic representation of minimal network miRNA involvement in gene regulation in cell pathways. Highlighted genes with symbols are involved in pathways in cancer.

Based on the FFPE miRNA expression data, we determined probable miRNA-3′UTR mRNA correlative interactions using an in silico centric network analysis. The miRTarBase database of experimentally validated miRNA-targets was used to determine these correlative interactions. The network analysis of DESeq2 normalized miRNA counts was performed on FFPE miRNA data, which provided a narrower set of differentially expressed miRNAs compared to FFT and included statistical significance with fold change information. Significantly expressed miRNAs between WT and HK tissue with FDR < 0.01 and |log2FoldChange| > 2 were considered in the pathway analysis (DESeq2 results). Based on the results of the basic analysis (17 queries, 3943 nodes and 5648 edges), a minimal network analysis was introduced, proposing a model with 17 variables, 103 nodes and 280 edges (Figure 3). The results of the hypergeometric test miRNA target analysis indicated 10 cellular pathways all associated with tumor pathogenesis (Table S8). The significantly regulated pathways included the following 14 genes: *AKT1*, *ATM*, *BCL2*, *CAV1*, *CCND1*, *CDKN1DIAPH1*, *ERBB2*, *FAS*, *HIF1A*, *IGF1R*, *ITGA3*, *MDM2* and *VAV3.* The pathways with the most affected genes reported in the analysis were Pathways in cancer, affected by 10 genes: *AKT1*, *BCL2*, *CCND1*, *CDKN1B*, *ERBB2*, *FAS*, *HIF1A*, *IGF1R*, *ITGA3* and *MDM2*. Most of the genes regulated by these miRNAs, with decreased expression in tumor tissues, are involved in cancer (Table 2).

**Figure 4:**
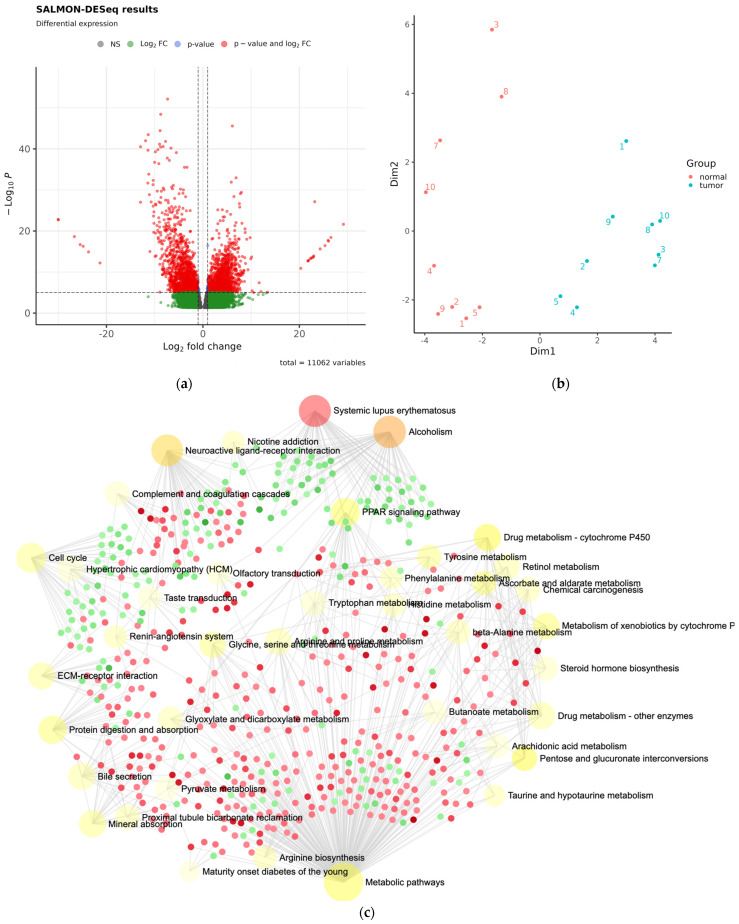
WT mRNA differential expression and mRNA pathway analysis. Differentially expressed mRNA results of FFT Wilms tumor compared to HK as presented by DESeq2 analysis as (**a**) volcano plot and (**b**) PCA plot (N = 9). (**c**) Graphical presentation of differentially expressed mRNA involved in cellular processes reported by the NetworkAnalyst. Red dots represent mRNAs with decreased expression; green dots show mRNAs with increased expression in WT. Red dots represent transcripts with decreased expression and green dots represent transcripts with increased expression.

To better define the function and hypothesized model of how miRNAs in WT affect the renal transcriptome, which was hypothesized on FFPE data, we performed mRNA sequencing of WT and HK FFT samples. Expression analysis revealed significant differences (|log2FoldChange| > 2, FDR < 0.05) reflected as a global change in the transcriptome with 7207 affected mRNA transcripts (Figure 4a,b; Supplementary Table S7). Differentially expressed mRNAs were associated with multiple cellular processes and pathological conditions, according to network analysis (NetworkAnalyst; DESeq2 normalized data) (Table 3, Figure 4c, Supplementary Table S7). Most differentially expressed mRNAs in WT compared to HK were involved in metabolic pathways, indicating decreased metabolic activity in WT tissues. In addition, WT tissues showed decreased mRNA expression, which is involved in drug metabolism and mineral absorption. In contrast, there was a significant increase in the expression of histone complex genes, which the KEGG database classifies as the occurrence of SLE and alcoholism, characterized by tissue inflammation, renal tissue damage and renal function impairment [21]. These pathways might be affected by cancer itself or WT therapy.

Cross-sectional comparison of 14 predicted mRNAs regulated by differentially expressed miRNAs identified by miRnet in silico minimal network pathway analysis (Supplementary Table S8) and mRNA sequencing confirmed the differential expression of only four mRNAs. *ERBB2*, *DIAPH1* and *VAV3* transcripts showed decreased expression whereas *FAS* expression was increased in the WT. According to miRNA pathway analysis, FAS protein is involved in cellular pathways involved in the p53 cancer signaling pathway and apoptosis, ERBB2 protein is involved in cellular pathways in cancer and bladder cancer, and DIAPH and VAV3 are involved in focal cell adhesion.

## 4. Discussion

**Figure 5:**
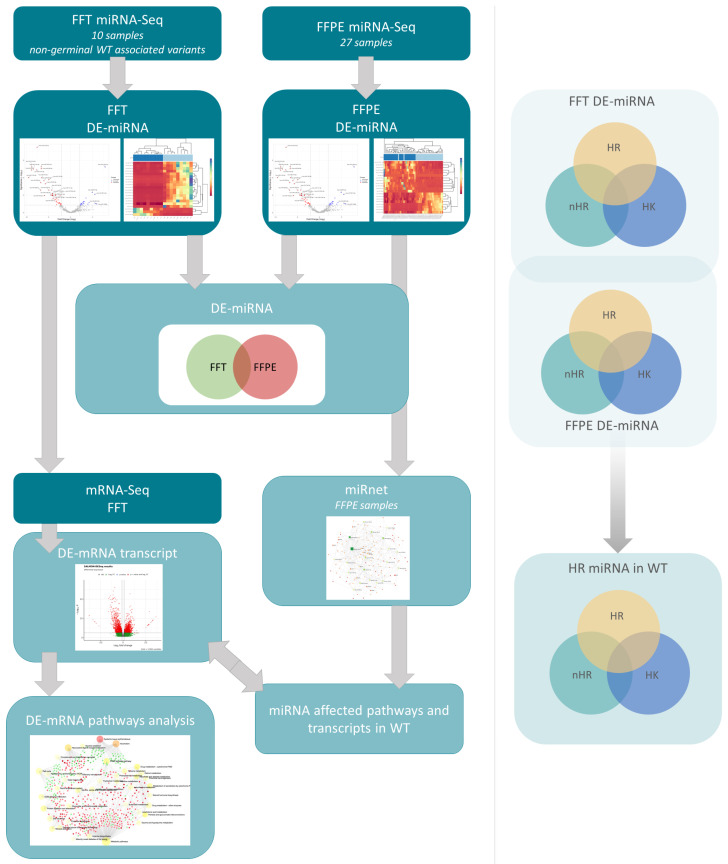
Study scheme. In our study, we analyzed miRNA expressions in WT FFT and FFPE samples to detect differentially expressed universal miRNAs, regardless of sample type and tumor histology. Additionally, differentially expressed miRNA expression in HR, nHR and HK were assessed. in silico network analysis of miRNAs with potentially affected transcripts was performed on FFPE miRNA expression data, which included fold change data and statistical significance. This data was compared with mRNA expression analysis and mRNA pathway analysis in FFT to show miRNA-mRNA correlation. Our study identified differentially expressed candidate miRNAs as potential and more suitable universal biomarkers compared to mRNAs.

The identification of differentially expressed mRNAs and miRNAs specific to WT tissue is a promising approach for developing new diagnostic and therapeutic strategies that may improve the prognosis and outcome of patients with the WT [1,9]. Our study aimed to establish a clinically relevant dataset of WT-related biomarkers that can be applied irrespective of the histological variants and disease stages (Figure 5). WT is a heterogeneous tumor comprising diverse components of the stroma, blastema, epithelium, anaplasia and regressive changes in the case of neoadjuvant chemotherapy [22].

NGS technologies have enabled the analysis of gene expression patterns with unprecedented levels of sensitivity and resolution, providing new opportunities for the identification of novel biomarkers in the Wilms tumor. This constituted the primary rationale for utilizing NGS, which, unlike other techniques such as qPCR and microarray, allows analyzing the entire miRNA and mRNA expression profile and predicting correlated interactions non-dependent on histological diversity and tumor stage.

A total of 74 kidney tissue samples obtained from 37 subjects were analyzed and divided into two independent groups of FFT and FFPE samples. Analysis of FFT samples from surgically removed kidneys identified miRNA candidates that exhibited significantly increased or decreased expression in WT compared to HK (Figure 1). Although the miRNA expression profiles in the FFT group were homogeneous, suggesting sample similarities, the number of biological replicates was limited. To address this limitation, we included an additional independent group of FFPE samples. We validated the results of the significantly expressed miRNAs in FFT samples by analyzing miRNA expression in 27 additional FFPE WT and matched HK tissue samples, where WT FFPE samples also showed similar miRNA profiles (Figure 2). Our tumor subtype and stage non-discriminatory study approach with two independent tumor groups resulted in 41 differentially expressed miRNAs, with 27 miRNAs showing decreased expression and 14 miRNAs with increased expression in WT compared to HK tissue (Table 1). Although some previous studies have investigated miRNAs in the WT using microarrays and qPCR methods, to our knowledge, our study is the first to use a comprehensive multi-omics NGS approach to determine a complete miRNA profile. Some of the differentially expressed miRNAs have already been reported to be differentially expressed in the WT, where miRNAs were investigated according to WT type or miRNAs were reported in subjects with the WT as cell-free blood plasma biomarkers [23,24,25]. Several studies have reported upregulated miR-483-5p and miR-483-3p in the WT, which were also increasingly expressed in our study [26,27] and have previously been described as being associated with increased proliferation, migration, invasion and decreased apoptosis of WT cells and high-risk WTs [26,27,28,29]. Upregulated miR-106-3p expression in the WT has been reported in blastemal WT samples [26], whereas increased expression of miR-130b-3 has also been described as a possible biomarker in the serum of WT patients [30].

miR-192, miR-194 and miR-215 have been reported to be downregulated in all WTs, regardless of subtype, while miR-200c and miR-141 are recognized as decreased in most WTs, except stromal and epithelial WT [31]. Previous studies have also demonstrated reduced expression of the entire miR-200 family in the WT, which has been linked to various processes, such as inhibition of metastasis, epithelial-mesenchymal transition and angiogenesis [32,33,34]. In this context, miR-200b-3p has been identified to be associated with metastatic WT [35], and our results indicate that miR-200b-3p is a potential universal WT miRNA. Recent studies have reported interesting results regarding the downregulation of miR-184 and miR-194-5p in both regressive and blastemal tumors [24,25]. Our findings support these results for differentially expressed miRNAs but put the identified miRNAs in the context of comprehensive markers of WT.

Many of the miRNAs differentially expressed in our study have been previously documented in tumors but have not been described in WT pathogenesis. miR-509-3-5p is a tumor suppressor that is downregulated in lung and gastric cancers [36,37]. miR-30c-2-3p is downregulated in clear cell renal cell carcinoma [38] while miR-138-5p and miR-139-5p suppress cell growth and invasion in many solid and hematological neoplasms [39,40]. miR-190a-5p expression is decreased in squamous cell lung carcinoma [41]. miR-378a-3p influences carcinogenesis of epithelial-mesenchymal transition [42], and miR-378d and miR-378f are downregulated in colorectal carcinoma [43]. Reports of miR-455-5p downregulation have been considered in prostate carcinoma, exposing its tumor suppressive function, inhibiting proliferation and triggering apoptosis [44].

In our study, we also tried to identify whether the miRNA profile can be used to determine whether WT is HR or nHR. We did not observe significant differences in miRNA expression between HR and nHR WT. This may be due to the small number of HK samples, which did not have the most homogeneous miRNA expression among them. In addition, we identified two miRNAs that are differentially expressed in HR compared to normal tissue and were not differentially expressed in nHR when these samples were compared to HK. However, these results are inconclusive due to the small number of HR samples compared.

To define the role of differentially expressed miRNAs in nephroblastoma, we performed in silico pathway analysis on FFPE sequencing data, which provided a smaller number of differentially expressed miRNA. This analysis revealed significant changes in cellular pathways and genes regulated by miRNAs, among which differentially expressed miRNAs regulated genes related to cancer and cancer-related pathways (Figure 3, Table 2). Although our minimal network analysis included the most differentially expressed miRNAs, previous WT studies incorporating bioinformatics network approaches and multidimensional integration strategies have additionally reported increased miR-34a, miR106-3p and miR-335 -5p in the WT [45,46], the expression of which was altered in our WT samples.

To confirm miRNA-mRNA correlative interactions suggested in silico analysis of FFPE data, we conducted mRNA sequencing of the independent samples of FFT nephroblastoma tissue. Differential mRNA analysis revealed a significant tumor profile with over 5105 differentially expressed mRNAs (Figure 4a), representing a substantial proportion of the changes in the overall transcriptional profile associated with highly altered transcriptional activity in cancer. Cross-sectional analysis of genes reported by the miRNA minimal network and differential mRNA expression confirmed a limited number of genes reported in the carcinogenesis of several cancers [47,48,49]. However, mRNAs were found to be less suitable as potential markers due to their globally altered transcriptomic profile, as supported by evidence from network analysis showing affected cellular pathways not directly associated with cancer, which is not entirely consistent with the previous by integrated bioinformatics study [50]. In contrast, miRNA pathway analysis revealed that miRNAs are involved in cancer-related pathways. Transcriptomic mRNA pathway analysis showed altered cellular and pathogenesis-associated processes in pathways related to chromatin activity, drug metabolism and cellular metabolism (Figure 4c). These differences in transcriptomic profiles are likely due to chemotherapy treatment and related tissue changes, as identified in the pathway analysis of the mRNAs involved in drug and cell metabolism (Table 3).

The limitation of our study may be the neoadjuvant chemotherapy received by patients according to the SIOP RTSG treatment protocol, which is used irrespective of the histological subtype and may affect cellular processes in the tumor tissue. Chemotherapy can induce tissue necrosis, fibrosis and metabolic changes. However, our samples did not exhibit a significant percentage of such changes, reflecting the homogeneity of the results. Even when detected in our samples, they did not significantly affect the overall diversity of the transcriptomic profiles in the studied groups, which was controlled and shown by the multiomic perspective of our study. Furthermore, miRNA transcriptome profiling of two independent study groups with different sample types, followed by whole transcriptome profiling, highlights the role of miRNAs in the WT, which is an advantage of this study. Although the limitation of our study is the small number of cases, an additional advantage of our study is two differently processed groups of samples (FFT and FFPE). This approach provides stable miRNA potential biomarkers and correlative miRNA-mRNA interactions, irrespective of the sample form and processing.

Based on our results, we conclude that miRNAs are better and more specific markers of WT than mRNAs, which gives miRNAs additional relevance for further validation as WT biomarkers. Thus, this study represents a basis for the identification of non-invasive biomarkers in WT diagnostics from urine, which can provide a diagnostic tool for tracking potentially malignant changes. With the increasing accessibility of human DNA sequencing and genomic screening, it will be possible to detect germline changes in the WT1 gene associated with an increased risk of developing Wilms tumor, as recommended by The American College of Medical Genetics and Genomics. Due to the non-invasive process of collecting urine samples, the potential applicability of detecting miRNAs in urine for diagnostic purposes has already been investigated in a number of diseases, including cancer [51,52]. In prostate cancer, miRNAs from urine extracellular vesicles have already been used as a supportive diagnostic tool [53]. Considering these facts, validated urine non-invasive miRNA early stage WT biomarkers, together with genetic predisposition testing, could establish a comprehensive clinical practice for longitudinal monitoring and early intervention in children at increased risk of the WT.

In addition, it would also be useful to determine the impact of somatic variants in relation to miRNA expression. Since miRNAs and their antisense sequences have been identified as potential therapeutic targets in numerous pathologies, our findings on differentially expressed miRNAs hold significant promise as potential targets for the treatment and prevention of the WT. The development of miRNA-targeting therapies for the WT could provide an effective approach for personalized treatment and improve patient outcomes, with the aim of preventing tumor development and preserving the affected kidneys [54].

## 5. Conclusions

Genetics, epigenetics such as miRNAs, and proteins involved in miRNA cellular processing play an important role in the development of the WT. In addition to the regulatory function of miRNAs, these molecules are also being studied as biological markers for non-invasive or minimally invasive primary diagnostic tools because of their amenability for rapid and cost-effective PCR detection. Our study identified 27 miRNAs with decreased expression and 14 miRNAs with increased expression in WT tissue. Additional multicenter evaluation of upregulated and downregulated suggested WT miRNAs will emphasize their potential as non-invasive biomarkers in urine or plasma and their therapeutic role in preserving the affected kidney and in preventing tumor pathogenesis in individuals with WT germinal genetic variants.

## Figures and Tables

**Table 1:** Downregulated and upregulated miRNAs in WT tissue. Based on both the FFT and FFPE miRNA differential expression analysis, we identified 27 miRNAs with decreased and 14 miRNAs with increased expression in WT tissue compared to HK tissue.

Decreased Expression in WT Tissue	Increased Expression in WT Tissue
hsa-miR-9-5p	hsa-miR-34a-5p
hsa-miR-30a-5p	hsa-miR-106b-3p
hsa-miR-30b-5p	hsa-miR-130b-3p
hsa-miR-30c-2-3p	hsa-miR-130b-5p
hsa-miR-138-5p	hsa-miR-181a-2-3p
hsa-miR-139-5p	hsa-miR-199b-5p
hsa-miR-141-3p	hsa-miR-206
hsa-miR-184	hsa-miR-323a-3p
hsa-miR-190a-5p	hsa-miR-335-3p
hsa-miR-192-5p	hsa-miR-335-5p
hsa-miR-194-5p	hsa-miR-483-3p
hsa-miR-200a-3p	hsa-miR-483-5p
hsa-miR-200a-5p	hsa-miR-873-5p
hsa-miR-200b-3p	hsa-miR-1269a
hsa-miR-200c-3p	
hsa-miR-204-5p	
hsa-miR-215-5p	
hsa-miR-378a-3p	
hsa-miR-378c	
hsa-miR-378d	
hsa-miR-378f	
hsa-miR-422a	
hsa-miR-429	
hsa-miR-455-5p	
hsa-miR-509-3-5p	
hsa-miR-514a-3p	
hsa-miR-12135	

**Table 2:** KEGG regulatory cellular pathways regulated by differentially expressed miRNAs in WT according to miRnet analysis (minimal network).

Name	Hits	*p* Value	*FDR*
Prostate cancer	7	0.00000394	0.000394
Focal adhesion	9	0.0000174	0.00087
Pathways in cancer	10	0.0000981	0.00327
Bladder cancer	3	0.00144	0.0269
Glioma	4	0.00159	0.0269
p53 signaling pathway	4	0.00188	0.0269
Melanoma	4	0.00188	0.0269
Chronic myeloid leukemia	4	0.00244	0.0305
Small cell lung cancer	4	0.00341	0.0379
Apoptosis	4	0.00389	0.0389

**Table 3:** mRNA KEGG cellular pathways involved in differentially expressed mRNAs in WT according to NetworkAnalyst.

Name	Hits (mRNA/Genes)	*p* Value	*FDR*
Systemic lupus erythematosus	54/66	1.96 × 10^−16^	6.2 × 10^−14^
Alcoholism	59/90	1.38 × 10^−10^	2.19 × 10^−8^
Neuroactive ligand–receptor interaction	45/71	1.1 × 10^−7^	1.17 × 10^−5^
Drug metabolism–cytochrome P450	18/22	3.31 × 10^−6^	2.62 × 10^−4^
Pentose and glucuronate interconversions	13/14	5.18 × 10^−6^	3.28 × 10^−4^
Metabolic pathways	236/593	4.97 × 10^−5^	0.00245
PPAR signaling pathway	27/43	5.42 × 10^−5^	0.00245
Metabolism of xenobiotics by cytochrome P450	15/21	3.47 × 10^−4^	0.0138
Ascorbate and aldarate metabolism	10/12	4.91 × 10^−4^	0.0173
Protein digestion and absorption	23/39	7.23 × 10^−4^	0.0229
Arginine and proline metabolism	18/29	0.00121	0.0348
Glycine, serine and threonine metabolism	17/27	0.00132	0.0348
Mineral absorption	19/32	0.00188	0.0459
Drug metabolism–other enzymes	18/30	0.0021	0.0476

## Data Availability

Data supporting the findings of this study are available in the Sequence Read Archive (SRA) repository: SUB13605381.

## Supplementary Materials

The following supporting information can be downloaded at: https://www.mdpi.com/article/10.3390/cancers15153846/s1, Table S1: Histological characteristics of FFT samples included in the analysis of differentially expressed miRNAs in fresh frozen tissue Wilms tumor samples; Table S2a–c: Differentially expressed miRNAs in FFT samples of WT identified using at least three differential expression tools (sRNAde tool). Table S2a shows decreased miRNA expression in WT; Table S2b shows increased miRNA expression in WT compared to normal renal tissue; and Table S2c presents DESeq2 differential expression results. Table S3: Histological characteristics of FFPE samples included in the analysis of differentially expressed miRNAs in formaldehyde-fixed paraffin-embedded tissue Wilms tumor samples. Table S4a–c: Differentially expressed miRNAs in FFPE samples of WT identified by at least three differential expression tools (sRNAde tool). Table S4a shows decreased miRNA expression in WT; Table S4b shows increased miRNA expression in WT compared to normal renal tissue; and Table S4c presents DESeq2 differential expression results. Table S5a,b: Differentially expressed miRNAs in FFT and FFPE HR, nHR and HK samples identified by at least three differential expression tools (sRNAde tool). Table S5a shows decreased miRNA expression, and Table S5b shows increased miRNA expression in compared sample groups (HR—High-Risk WT, nHR—non-High-Risk WT; HK—Healthy Kidney). Table S6: Cross-sectional analysis of FFT and FFPE differential expression miRNAs of HR, nHR and HR WT samples. We did not identify significant miRNAs for HR WT when HR samples were compared to nHR. In contrast, by comparing HR WT and nHR WT with HK samples, we identified 5 miRNAs with decreased expression and 6 miRNAs with increased expression in HR WT. Figure S1: Presentation of the similarity of the compared samples by principal component analysis of HR-nHR-HK DESeq2 miRNA differential expression FFT and FFPE samples. HR tumor samples are marked in green, nHR tumor samples in orange and HK tumor samples in purple. Table S7: mRNA DESeq2 results. Table S8: Predicted mRNAs regulated by differentially expressed miRNAs identified by miRnet in silico minimal network pathway analysis. Highlighted mRNA transcripts in red represent differentially expressed transcripts in the WT, as confirmed by mRNA sequencing analysis.

## References

[1] Leichter A.L., Sullivan M.J., Eccles M.R., Chatterjee A. (2017). MicroRNA Expression Patterns and Signalling Pathways in the Development and Progression of Childhood Solid Tumours. Mol. Cancer.

[2] Treger T.D., Chowdhury T., Pritchard-Jones K., Behjati S. (2019). The Genetic Changes of Wilms Tumour. Nat. Rev. Nephrol..

[3] Maciaszek J.L., Oak N., Nichols K.E. (2020). Recent Advances in Wilms’ Tumor Predisposition. Hum. Mol. Genet..

[4] Smolarz B., Durczyński A., Romanowicz H., Szyłło K., Hogendorf P. (2022). MiRNAs in Cancer (Review of Literature). Int. J. Mol. Sci..

[5] Cerqueira D.M., Tayeb M., Ho J. (2022). MicroRNAs in Kidney Development and Disease. JCI Insight.

[6] Du T., Zamore P.D. (2005). MicroPrimer: The Biogenesis and Function of MicroRNA. Development.

[7] Ohtsuka M., Ling H., Doki Y., Mori M., Calin G.A. (2015). MicroRNA Processing and Human Cancer. J. Clin. Med..

[8] Lu J., Getz G., Miska E.A., Alvarez-Saavedra E., Lamb J., Peck D., Sweet-Cordero A., Ebert B.L., Mak R.H., Ferrando A.A. (2005). MicroRNA Expression Profiles Classify Human Cancers. Nature.

[9] Salomão K.B., Pezuk J.A., de Souza G.R., Chagas P., Pereira T.C., Valera E.T., Brassesco M.S. (2019). MicroRNA Dysregulation Interplay with Childhood Abdominal Tumors. Cancer Metastasis Rev..

[10] Brok J., Treger T.D., Gooskens S.L., van den Heuvel-Eibrink M.M., Pritchard-Jones K. (2016). Biology and Treatment of Renal Tumours in Childhood. Eur. J. Cancer.

[11] Li Y., Tang H., Huang Z., Qin H., Cen Q., Meng F., Huang L., Lin L., Pu J., Yang D. (2022). Bioinformatics Analysis and Identification of Genes and Pathways Involved in Patients with Wilms Tumor. Transl. Cancer Res..

[12] Chapman B., Kirchner R., Pantano L., Naumenko S., De Smet M., Beltrame L., Khotiainsteva T., Sytchev I., Guimera R.V., Kern J. bcbio/bcbio-nextgen: v1.2.5 (v1.2.5). Zenodo 2021. https://zenodo.org/record/5781867.

[13] Li H., Durbin R. (2009). Fast and Accurate Short Read Alignment with Burrows-Wheeler Transform. Bioinformatics.

[14] Desvignes J.P., Bartoli M., Delague V., Krahn M., Miltgen M., Béroud C., Salgado D. (2018). VarAFT: A Variant Annotation and Filtration System for Human next Generation Sequencing Data. Nucleic. Acids Res..

[15] Aparicio-Puerta E., Gómez-Martín C., Giannoukakos S., Medina J.M., Scheepbouwer C., García-Moreno A., Carmona-Saez P., Fromm B., Pegtel M., Keller A. (2022). SRNAbench and SRNAtoolbox 2022 Update: Accurate MiRNA and SncRNA Profiling for Model and Non-Model Organisms. Nucleic. Acids Res..

[16] Chang L., Xia J. (2023). MicroRNA Regulatory Network Analysis Using MiRNet 2.0. Methods Mol. Biol..

[17] Bhardwaj V., Heyne S., Sikora K., Rabbani L., Rauer M., Kilpert F., Richter A.S., Ryan D.P., Manke T. (2019). SnakePipes: Facilitating Flexible, Scalable and Integrative Epigenomic Analysis. Bioinformatics.

[18] Martin M. (2011). Cutadapt Removes Adapter Sequences From High-Throughput Sequencing Reads. EMBnet. J..

[19] Patro R., Duggal G., Love M.I., Irizarry R.A., Kingsford C. (2017). Salmon Provides Fast and Bias-Aware Quantification of Transcript Expression. Nat. Methods.

[20] Love M.I., Huber W., Anders S. (2014). Moderated Estimation of Fold Change and Dispersion for RNA-Seq Data with DESeq2. Genome Biol..

[21] Wang Z., Chang C., Peng M., Lu Q. (2017). Translating Epigenetics into Clinic: Focus on Lupus. Clin. Epigenetics.

[22] Vujanić G.M., Sandstedt B. (2010). The Pathology of Wilms’ Tumour (Nephroblastoma): The International Society of Paediatric Oncology Approach. J. Clin. Pathol..

[23] Ludwig N., Werner T.V., Backes C., Trampert P., Gessler M., Keller A., Lenhof H.P., Graf N., Meese E. (2016). Combining MiRNA and MRNA Expression Profiles in Wilms Tumor Subtypes. Int. J. Mol. Sci..

[24] Buglyó G., Magyar Z., Romicsné Görbe É., Bánusz R., Csóka M., Micsik T., Berki Z., Varga P., Sápi Z., Nagy B. (2019). Quantitative RT-PCR-Based MiRNA Profiling of Blastemal Wilms’ Tumors from Formalin-Fixed Paraffin-Embedded Samples. J. Biotechnol..

[25] Buglyó G., Magyar Z., Görbe É.R., Bánusz R., Csóka M., Micsik T., Mezei M., Yani J.A.S., Varga P., Sápi Z. (2021). MiRNA Profiling of Hungarian Regressive Wilms’ Tumor Formalin-Fixed Paraffin-Embedded (FFPE) Samples by Quantitative Real-Time Polymerase Chain Reaction (RT-PCR). Med. Sci. Monit..

[26] Watson J.A., Bryan K., Williams R., Popov S., Vujanic G., Coulomb A., Boccon-Gibod L., Graf N., Pritchard-Jones K., O’Sullivan M. (2013). MiRNA Profiles as a Predictor of Chemoresponsiveness in Wilms’ Tumor Blastema. PLoS ONE.

[27] Veronese A., Lupini L., Consiglio J., Visone R., Ferracin M., Fornari F., Zanesi N., Alder H., D’Elia G., Gramantieri L. (2010). Oncogenic Role of MiR-483-3p at the IGF2/483 Locus. Cancer Res..

[28] Liu M., Roth A., Yu M., Morris R., Bersani F., Rivera M.N., Lu J., Shioda T., Vasudevan S., Ramaswamy S. (2013). The IGF2 Intronic MiR-483 Selectively Enhances Transcription from IGF2 Fetal Promoters and Enhances Tumorigenesis. Genes Dev..

[29] Che G., Gao H., Tian J., Hu Q., Xie H., Zhang Y. (2020). MicroRNA-483-3p Promotes Proliferation, Migration, and Invasion and Induces Chemoresistance of Wilms’ Tumor Cells. Pediatr. Dev. Pathol..

[30] Hu Y., Yan J. (2019). Aberrant Expression and Mechanism of MiR-130b-3p/Phosphatase and Tensin Homolog in Nephroblastoma in Children. Exp. Ther. Med..

[31] Senanayake U., Das S., Vesely P., Alzoughbi W., Fröhlich L.F., Chowdhury P., Leuschner I., Hoefler G., Guertl B. (2012). MiR-192, MiR-194, MiR-215, MiR-200c and MiR-141 Are Downregulated and Their Common Target ACVR2B Is Strongly Expressed in Renal Childhood Neoplasms. Carcinogenesis.

[32] Li T., Zhao P., Li Z., Wang C.C., Wang Y.L., Gu Q. (2019). MiR-200c-3p Suppresses the Proliferative, Migratory, and Invasive Capacities of Nephroblastoma Cells via Targeting FRS2. Biopreserv. Biobank..

[33] Cao J., Liu G.-S., Zou N.-Z., Zhang H., He X.-X., Sun P.-L., An H.-J., Shen H. (2022). MicroRNA-200c-3p Suppresses Proliferation and Invasion of Nephroblastoma Cells by Targeting EP300 and Inactivating the AKT/FOXO1/P27 Pathway. Neoplasma.

[34] Pecot C.V., Rupaimoole R., Yang D., Akbani R., Ivan C., Lu C., Wu S., Han H.D., Shah M.Y., Rodriguez-Aguayo C. (2013). Tumour Angiogenesis Regulation by the MiR-200 Family. Nat. Commun..

[35] Pérez-Linares F.J., Pérezpeña-Diazconti M., García-Quintana J., Baay-Guzmán G., Cabrera-Muñoz L., Sadowinski-Pine S., Serrano-Bello C., Murillo-Maldonado M., Contreras-Ramos A., Eguía-Aguilar P. (2020). MicroRNA Profiling in Wilms Tumor: Identification of Potential Biomarkers. Front. Pediatr..

[36] Zhu Z., Yu Z., Rong Z., Luo Z., Zhang J., Qiu Z., Huang C. (2019). The Novel GINS4 Axis Promotes Gastric Cancer Growth and Progression by Activating Rac1 and CDC42. Theranostics.

[37] Liang J.-J., Wang J.-Y., Zhang T.-J., An G.-S., Ni J.-H., Li S.-Y., Jia H.-T. (2020). MiR-509-3-5p-NONHSAT112228.2 Axis Regulates P21 and Suppresses Proliferation and Migration of Lung Cancer Cells. Curr. Top. Med. Chem..

[38] Moch H., Lukamowicz-Rajska M. (2014). MiR-30c-2-3p and MiR-30a-3p: New Pieces of the Jigsaw Puzzle in HIF2α Regulation. Cancer Discov..

[39] Sukocheva O.A., Liu J., Neganova M.E., Beeraka N.M., Aleksandrova Y.R., Manogaran P., Grigorevskikh E.M., Chubarev V.N., Fan R. (2022). Perspectives of Using MicroRNA-Loaded Nanocarriers for Epigenetic Reprogramming of Drug Resistant Colorectal Cancers. Semin. Cancer Biol..

[40] Ghafouri-Fard S., Khoshbakht T., Hussen B.M., Taheri M., Samadian M. (2022). A Review on the Role of MCM3AP-AS1 in the Carcinogenesis and Tumor Progression. Cancer Cell Int..

[41] Wu D., Huo C., Jiang S., Huang Y., Fang X., Liu J., Yang M., Ren J., Xu B., Liu Y. (2021). Exostosin1 as a Novel Prognostic and Predictive Biomarker for Squamous Cell Lung Carcinoma: A Study Based on Bioinformatics Analysis. Cancer Med..

[42] Ghafouri-Fard S., Dashti S., Farsi M., Hussen B.M., Taheri M. (2021). A Review on the Role of Oncogenic LncRNA OIP5-AS1 in Human Malignancies. Biomed. Pharmacother..

[43] Gungormez C., Gumushan Aktas H., Dilsiz N., Borazan E. (2019). Novel MiRNAs as Potential Biomarkers in Stage II Colon Cancer: Microarray Analysis. Mol. Biol. Rep..

[44] Xing Q., Xie H., Zhu B., Sun Z., Huang Y. (2019). MiR-455-5p Suppresses the Progression of Prostate Cancer by Targeting CCR5. Biomed. Res. Int..

[45] He J., Guo X., Sun L., Wang K., Yao H. (2016). Networks Analysis of Genes and MicroRNAs in Human Wilms’ Tumors. Oncol. Lett..

[46] Chen W., Zhuang J., Gong L., Dai Y., Diao H. (2019). Investigating the Dysfunctional Pathogenesis of Wilms’ Tumor through a Multidimensional Integration Strategy. Ann. Transl. Med..

[47] Cuadrado M., Robles-Valero J. (2021). VAV Proteins as Double Agents in Cancer: Oncogenes with Tumor Suppressor Roles. Biology.

[48] Miao S., Schäfer P., Nojszewski J., Meyer F., Windhorst S. (2021). DIAPH1 Regulates Chromosomal Instability of Cancer Cells by Controlling Microtubule Dynamics. Eur. J. Cell Biol..

[49] Idossa D., Borrero M., Blaes A. (2023). ERBB2-Low (Also Known as HER2-Low) Breast Cancer. JAMA Oncol..

[50] Zhang L., Gao X., Zhou X., Qin Z., Wang Y., Li R., Tang M., Wang W., Zhang W. (2019). Identification of Key Genes and MicroRNAs Involved in Kidney Wilms Tumor by Integrated Bioinformatics Analysis. Exp. Ther. Med..

[51] Juracek J., Madrzyk M., Stanik M., Slaby O. (2022). Urinary MicroRNAs and Their Significance in Prostate Cancer Diagnosis: A 5-Year Update. Cancers.

[52] Aftab M., Poojary S.S., Seshan V., Kumar S., Agarwal P., Tandon S., Zutshi V., Das B.C. (2021). Urine MiRNA Signature as a Potential Non-Invasive Diagnostic and Prognostic Biomarker in Cervical Cancer. Sci. Rep..

[53] Kretschmer A., Kajau H., Margolis E., Tutrone R., Grimm T., Trottmann M., Stief C., Stoll G., Fischer C.A., Flinspach C. (2022). Validation of a CE-IVD, Urine Exosomal RNA Expression Assay for Risk Assessment of Prostate Cancer Prior to Biopsy. Sci. Rep..

[54] Hanna J., Hossain G.S., Kocerha J. (2019). The Potential for MicroRNA Therapeutics and Clinical Research. Front. Genet..

