# Supplementary material for: Integrative Transcriptomic Profiling of the Wilms Tumor
Source: Cancers (Basel). 2023 Jul 28;15(15):3846. doi: 10.3390/cancers15153846 (PMC10416970; doi:10.3390/cancers15153846)

Supplementary:
INTEGRATIVE TRANSCRIPTOMIC PROFILING OF WILMS TUMOR

Supplementary Figure S1: Presentation of the similarity of the compared samples by principal component analysis of HR-nHR-HK DESeq2 miRNA differential expression a) FFT and b) FFPE samples. HR tumor samples are marked in green, nHR tumor samples in orange and HK tumor samples in purple.

a

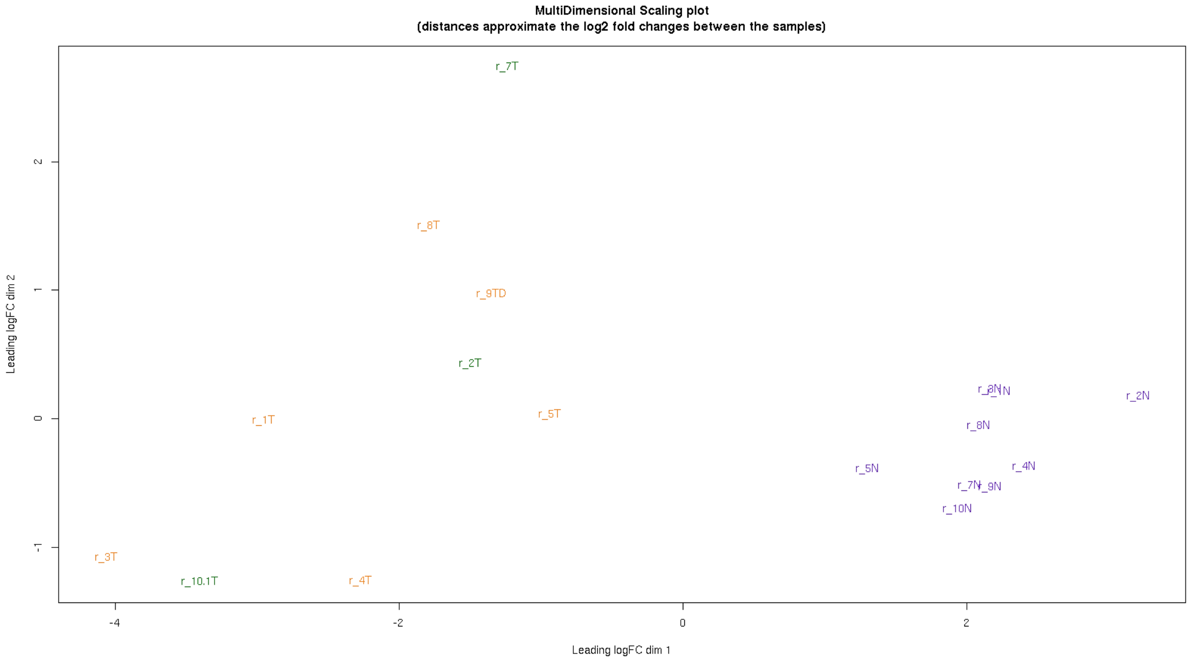

b

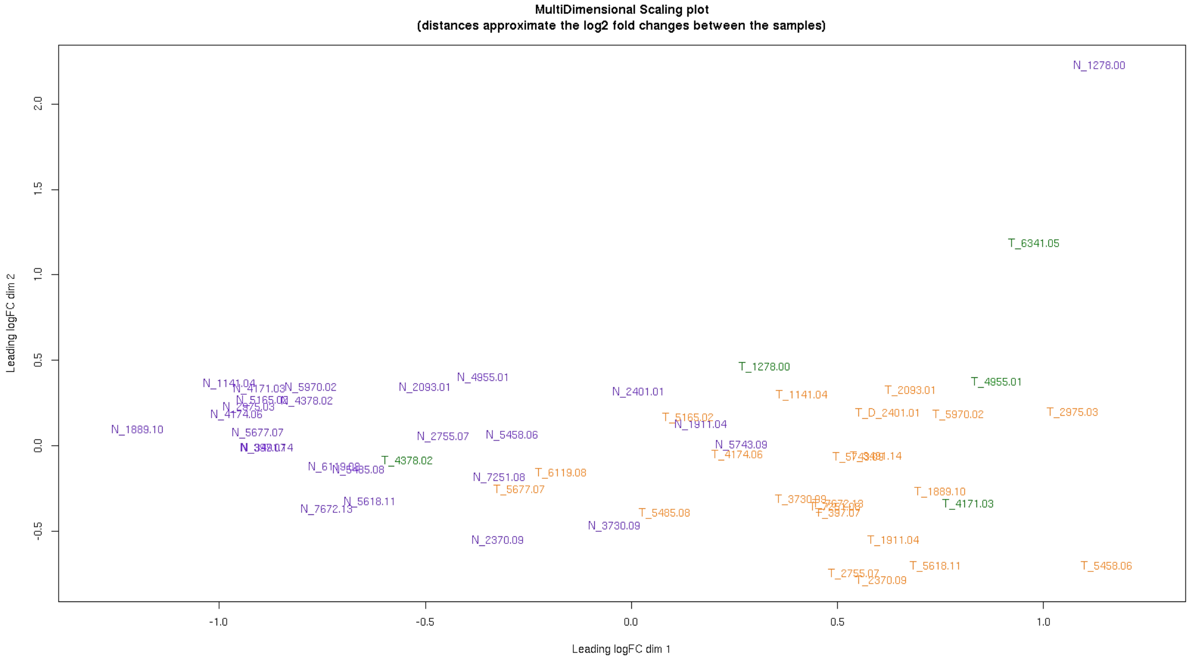

Supplement: Supplementary file 1 [file cancers-15-03846-s001.zip › Supplementary Figure S1.pdf]
