# Supplementary material for: Integrative Transcriptomic Profiling of the Wilms Tumor
Source: Cancers (Basel). 2023 Jul 28;15(15):3846. doi: 10.3390/cancers15153846 (PMC10416970; doi:10.3390/cancers15153846)
Supplement: Supplementary file 1 [file cancers-15-03846-s001.zip › Supplementary Table S1-S6, S8.pdf]

Supplementary:
INTEGRATIVE TRANSCRIPTOMIC PROFILING OF WILMS TUMOR

Supplementary Table S1: Histological characteristics of FFT samples included in the analysis of differentially expressed miRNAs in fresh frozen tissue Wilms tumor samples.

Patient No.	Healthy Tissue No.	Tumor Tissue No.	FFT tumor histology	***HR/nHR***	***Local WT sample Stage***
1	1N	1T	50% B, 35% E, 10% S, 5% R	nHR	III
2	2N	2T	60% E, 25% B, 5% R, 5% S, 5% A	**HR**	**I**
3	3N	3T	95% B, 5% E	nHR	I
4	4N	4T	45%S, 45% E, 10% HK	nHR	I
5	5N	5T	75% E, 25% S	nHR	I
6	6N	6T	75% TNe, 10% S, 10% R, 3-5% E	nHR	II
7	7N	7T	100 % B	**HR**	**III**
8	8N	8T	60% B, 40% S	nHR	III
9	9N	9T	40% R, 30% B, 30% E	nHR	I
10	10N	10T	90% B, 10%E	**HR**	**III**

N, healthy kidney tissue; T, Wilms tumor; FFT, fresh frozen tissue; %, the percentage of each histology component; S, stromal; R, regressive; B, blastemal; E, epithelial; A, anaplasia; TNe, tumor necrosis; HK, healthy kidney tissue. Sample 6 – necrosis, not included in further analysis. No malignant changes were detected in healthy tissue samples. High-Risk (HR), non-High-Risk (nHR) and local WT sample stage were assessed by the SIOP RTSG pathology protocol.

Supplementary:
INTEGRATIVE TRANSCRIPTOMIC PROFILING OF WILMS TUMOR

Supplementary Table S2: Differentially expressed miRNAs in FFT samples of WT identified using at least three differential expression tools (sRNAde tool). Table S2a) shows decreased miRNA expression in WT, and Table S2b) shows increased miRNA expression in WT compared to normal renal tissue.

S2a) Decreased expression of FFT miRNA	*No. DE Tools*	*DE tools*
hsa-miR-9-5p	5	DESeq,NOISeq,Students t-test,DESeq2,edgeR
hsa-miR-514a-5p	5	DESeq,NOISeq,Students t-test,DESeq2,edgeR
hsa-miR-514a-3p	5	DESeq,NOISeq,Students t-test,DESeq2,edgeR
hsa-miR-509-5p	5	DESeq,NOISeq,Students t-test,DESeq2,edgeR
hsa-miR-509-3p	5	DESeq,NOISeq,Students t-test,DESeq2,edgeR
hsa-miR-509-3-5p	5	DESeq,NOISeq,Students t-test,DESeq2,edgeR
hsa-miR-508-3p	5	DESeq,NOISeq,Students t-test,DESeq2,edgeR
hsa-miR-506-3p	5	DESeq,NOISeq,Students t-test,DESeq2,edgeR
hsa-miR-30c-2-3p	5	DESeq,NOISeq,Students t-test,DESeq2,edgeR
hsa-miR-30a-5p	5	DESeq,NOISeq,Students t-test,DESeq2,edgeR
hsa-miR-217-5p	5	DESeq,NOISeq,Students t-test,DESeq2,edgeR
hsa-miR-216b-5p	5	DESeq,NOISeq,Students t-test,DESeq2,edgeR
hsa-miR-215-5p	5	DESeq,NOISeq,Students t-test,DESeq2,edgeR
hsa-miR-204-5p	5	DESeq,NOISeq,Students t-test,DESeq2,edgeR
hsa-miR-203a-3p	5	DESeq,NOISeq,Students t-test,DESeq2,edgeR
hsa-miR-200b-3p	5	DESeq,NOISeq,Students t-test,DESeq2,edgeR
hsa-miR-200a-5p	5	DESeq,NOISeq,Students t-test,DESeq2,edgeR
hsa-miR-200a-3p	5	DESeq,NOISeq,Students t-test,DESeq2,edgeR
hsa-miR-194-5p	5	DESeq,NOISeq,Students t-test,DESeq2,edgeR
hsa-miR-194-3p	5	DESeq,NOISeq,Students t-test,DESeq2,edgeR
hsa-miR-192-5p	5	DESeq,NOISeq,Students t-test,DESeq2,edgeR
hsa-miR-190a-5p	5	DESeq,NOISeq,Students t-test,DESeq2,edgeR
hsa-miR-187-3p	5	DESeq,NOISeq,Students t-test,DESeq2,edgeR
hsa-miR-184	5	DESeq,NOISeq,Students t-test,DESeq2,edgeR
hsa-miR-141-3p	5	DESeq,NOISeq,Students t-test,DESeq2,edgeR
hsa-miR-139-5p	5	DESeq,NOISeq,Students t-test,DESeq2,edgeR
hsa-miR-139-3p	5	DESeq,NOISeq,Students t-test,DESeq2,edgeR
hsa-miR-12135	5	DESeq,NOISeq,Students t-test,DESeq2,edgeR
hsa-miR-934	4	DESeq,NOISeq,DESeq2,edgeR
hsa-miR-891a-5p	4	DESeq,Students t-test,DESeq2,edgeR
hsa-miR-6500-3p	4	DESeq,Students t-test,DESeq2,edgeR
hsa-miR-5588-5p	4	DESeq,Students t-test,DESeq2,edgeR
hsa-miR-514b-5p	4	DESeq,Students t-test,DESeq2,edgeR
hsa-miR-514b-3p	4	DESeq,Students t-test,DESeq2,edgeR
hsa-miR-513a-5p	4	DESeq,Students t-test,DESeq2,edgeR
hsa-miR-510-5p	4	DESeq,Students t-test,DESeq2,edgeR
hsa-miR-508-5p	4	DESeq,NOISeq,DESeq2,edgeR
hsa-miR-429	4	NOISeq,Students t-test,DESeq2,edgeR
hsa-miR-378a-3p	4	NOISeq,Students t-test,DESeq2,edgeR
hsa-miR-362-5p	4	NOISeq,Students t-test,DESeq2,edgeR
hsa-miR-30b-5p	4	NOISeq,Students t-test,DESeq2,edgeR
hsa-miR-23a-5p	4	DESeq,Students t-test,DESeq2,edgeR
hsa-miR-22-3p	4	NOISeq,Students t-test,DESeq2,edgeR
hsa-miR-216a-5p	4	DESeq,Students t-test,DESeq2,edgeR
hsa-miR-216a-3p	4	DESeq,Students t-test,DESeq2,edgeR
hsa-miR-200c-3p	4	NOISeq,Students t-test,DESeq2,edgeR
hsa-miR-192-3p	4	DESeq,Students t-test,DESeq2,edgeR
hsa-miR-141-5p	4	DESeq,Students t-test,DESeq2,edgeR
hsa-miR-138-5p	4	DESeq,Students t-test,DESeq2,edgeR
hsa-miR-660-3p	3	Students t-test,DESeq2,edgeR
hsa-miR-532-5p	3	Students t-test,DESeq2,edgeR
hsa-miR-532-3p	3	Students t-test,DESeq2,edgeR
hsa-miR-502-5p	3	Students t-test,DESeq2,edgeR
hsa-miR-500b-5p	3	Students t-test,DESeq2,edgeR
hsa-miR-497-5p	3	Students t-test,DESeq2,edgeR
hsa-miR-4802-3p	3	Students t-test,DESeq2,edgeR
hsa-miR-455-5p	3	Students t-test,DESeq2,edgeR
hsa-miR-422a	3	Students t-test,DESeq2,edgeR
hsa-miR-378i	3	Students t-test,DESeq2,edgeR
hsa-miR-378h	3	Students t-test,DESeq2,edgeR
hsa-miR-378g	3	Students t-test,DESeq2,edgeR
hsa-miR-378f	3	Students t-test,DESeq2,edgeR

hsa-miR-378e	3	Students t-test,DESeq2,edgeR
hsa-miR-378d	3	Students t-test,DESeq2,edgeR
hsa-miR-378c	3	Students t-test,DESeq2,edgeR
hsa-miR-378a-5p	3	Students t-test,DESeq2,edgeR
hsa-miR-3664-3p	3	Students t-test,DESeq2,edgeR
hsa-miR-365b-3p	3	Students t-test,DESeq2,edgeR
hsa-miR-365a-3p	3	Students t-test,DESeq2,edgeR
hsa-miR-30d-5p	3	Students t-test,DESeq2,edgeR
hsa-miR-30d-3p	3	Students t-test,DESeq2,edgeR
hsa-miR-30c-5p	3	Students t-test,DESeq2,edgeR
hsa-miR-30a-3p	3	Students t-test,DESeq2,edgeR
hsa-miR-29c-5p	3	Students t-test,DESeq2,edgeR
hsa-miR-29c-3p	3	Students t-test,DESeq2,edgeR
hsa-miR-29b-3p	3	Students t-test,DESeq2,edgeR
hsa-miR-29b-1-5p	3	Students t-test,DESeq2,edgeR
hsa-miR-29a-5p	3	Students t-test,DESeq2,edgeR
hsa-miR-29a-3p	3	Students t-test,DESeq2,edgeR
hsa-miR-22-5p	3	Students t-test,DESeq2,edgeR
hsa-miR-211-5p	3	Students t-test,DESeq2,edgeR
hsa-miR-204-3p	3	Students t-test,DESeq2,edgeR
hsa-miR-203b-3p	3	Students t-test,DESeq2,edgeR
hsa-miR-200b-5p	3	Students t-test,DESeq2,edgeR
hsa-miR-145-5p	3	Students t-test,DESeq2,edgeR
hsa-miR-126-5p	3	Students t-test,DESeq2,edgeR
hsa-miR-126-3p	3	Students t-test,DESeq2,edgeR
hsa-miR-12136	3	NOISeq,DESeq2,edgeR

S2b) Increased expression of FFT miRNA	*No. DE Tools*	*DE tools*
hsa-miR-135a-3p	5	DESeq,NOISeq,Students t-test,DESeq2,edgeR
hsa-miR-199b-5p	5	DESeq,NOISeq,Students t-test,DESeq2,edgeR
hsa-miR-106b-3p	4	DESeq,Students t-test,DESeq2,edgeR
hsa-miR-1269a	4	DESeq,NOISeq,DESeq2,edgeR
hsa-miR-130b-3p	4	DESeq,NOISeq,DESeq2,edgeR
hsa-miR-130b-5p	4	DESeq,NOISeq,DESeq2,edgeR
hsa-miR-181b-2-3p	4	DESeq,Students t-test,DESeq2,edgeR
hsa-miR-18a-5p	4	DESeq,Students t-test,DESeq2,edgeR
hsa-miR-301b-3p	4	DESeq,NOISeq,DESeq2,edgeR
hsa-miR-6818-5p	4	DESeq,Students t-test,DESeq2,edgeR
hsa-miR-873-3p	4	DESeq,NOISeq,DESeq2,edgeR
hsa-miR-873-5p	4	DESeq,Students t-test,DESeq2,edgeR
hsa-let-7d-5p	3	Students t-test,DESeq2,edgeR
hsa-let-7e-3p	3	Students t-test,DESeq2,edgeR
hsa-miR-1304-5p	3	Students t-test,DESeq2,edgeR
hsa-miR-17-5p	3	Students t-test,DESeq2,edgeR
hsa-miR-181a-2-3p	3	Students t-test,DESeq2,edgeR
hsa-miR-181b-3p	3	DESeq,DESeq2,edgeR
hsa-miR-182-5p	3	NOISeq,DESeq2,edgeR
hsa-miR-183-5p	3	NOISeq,DESeq2,edgeR
hsa-miR-196a-5p	3	NOISeq,DESeq2,edgeR
hsa-miR-206	3	NOISeq,DESeq2,edgeR
hsa-miR-20a-3p	3	Students t-test,DESeq2,edgeR
hsa-miR-20a-5p	3	Students t-test,DESeq2,edgeR
hsa-miR-301a-3p	3	DESeq,DESeq2,edgeR
hsa-miR-323a-3p	3	DESeq,DESeq2,edgeR
hsa-miR-335-3p	3	DESeq,DESeq2,edgeR
hsa-miR-335-5p	3	Students t-test,DESeq2,edgeR
hsa-miR-34a-5p	3	Students t-test,DESeq2,edgeR
hsa-miR-374b-3p	3	Students t-test,DESeq2,edgeR
hsa-miR-412-5p	3	DESeq,DESeq2,edgeR
hsa-miR-433-3p	3	NOISeq,DESeq2,edgeR
hsa-miR-4443	3	Students t-test,DESeq2,edgeR
hsa-miR-4738-3p	3	Students t-test,DESeq2,edgeR
hsa-miR-483-3p	3	NOISeq,DESeq2,edgeR
hsa-miR-483-5p	3	NOISeq,DESeq2,edgeR
hsa-miR-887-5p	3	Students t-test,DESeq2,edgeR
hsa-miR-93-5p	3	DESeq,DESeq2,edgeR
hsa-miR-96-5p	3	NOISeq,DESeq2,edgeR

Supplementary:
INTEGRATIVE TRANSCRIPTOMIC PROFILING OF WILMS TUMOR

Supplementary Table S3: Histological characteristics of FFPE samples included in the analysis of differentially expressed miRNAs in formaldehyde-fixed paraffin-embedded tissue Wilms tumor samples.

Patient No.	*Healthy Tissue No.*	*Tumor Tissue No.*	*FFPE tumor histology*	*HR/nHR*	*Local WT sample Stage*
1	1278/00 N	1278/00 T	100% B	**HR**	III
2	2093/01 N	2093/01 T	50% B, 50% E	nHR	III
3	2401/01 N	2401/01 T	50% S, 45% E, 5% B	nHR	I
4	4955/01 N	4955/01 T	70% S, 30% B	**HR**	I
5	4378/02 N	4378/02 T	95% B, 5% R	**HR**	II
6	5165/02 N	5165/02 T	95% B, 5% R	nHR	I
7	5970/02 N	5970/02 T	50% S, 45% E, 5% B	nHR	III
8	2975/03 N	2975/03 T	90% S, 10% R	NHR	I
9	4171/03 N	4171/03 T	70% B, 20% S, 10% E	**HR**	II
10	1141/04 N	1141/04 T	90% E, 10% R	nHR	I
11	1911/04 N	1911/04 T	70% S, 25% E, 5% B	nHR	I
12	6341/05 N	6341/05 T	100% B	**HR**	I
13	4174/06 N	4174/06 T	80% E, 20% S	nHR	I
14	5458/06 N	5458/06 T	80% S, 15% B, 5% E	nHR	II
15	397/07 N	397/07 T	70% E, 30% S	nHR	I
16	2755/07 N	2755/07 T	45% B, 45% E, 10% S	nHR	I
17	5677/07 N	5677/07 T	100% E	nHR	I
18	5485/08 N	5485/08 T	75% E, 15% S, 10% B	nHR	II
19	6119/08 N	6119/08 T	100% E	nHR	I
20	7251/08 N	7251/08 T	60% S, 40% E	nHR	II
21	2370/09 N	2370/09 T	80% E, 15% S, 5% B	nHR	I
22	3730/09 N	3730/09 T	80% B, 20% E	nHR	I
23	5743/09 N	5743/09 T	95% E, 5% S	nHR	I
24	1889/10 N	1889/10 T	75% E, 25% R	nHR	II
25	5618/11 N	5618/11 T	50% E, 50% S	nHR	I
26	7672/13 N	7672/13 T	60% S, 40% B	nHR	III
27	3491/14 N	3491/14 T	80%S,10% R, 5% E, 5% B	nHR	I

N-healthy kidney tissue, T-WT, %-percentage of each histology component, S-stromal, R-regressive, B-blastemal, E-epithelial. High-Risk (HR), non-High-Risk (nonHR) and local WT sample stage were assessed by the SIOP RTSG pathology protocol.

Supplementary:
INTEGRATIVE TRANSCRIPTOMIC PROFILING OF WILMS TUMOR

Supplementary Table S4 A, B: Differentially expressed miRNAs in FFPE samples of WT identified by at least three differential expression tools (sRNAde tool). Table 4Sa) shows decreased miRNA expression in WT, and Table 4Sb) shows increased miRNA expression in WT compared to normal renal tissue.

S4a) Decreased expression of FFPE miRNA	*No. DE Tools*	*DE tools*
hsa-miR-12135	5	DESeq,NOISeq,Students t-test,DESeq2,edgeR
hsa-miR-192-5p	5	DESeq,NOISeq,Students t-test,DESeq2,edgeR
hsa-miR-194-5p	5	DESeq,NOISeq,Students t-test,DESeq2,edgeR
hsa-miR-215-5p	5	DESeq,NOISeq,Students t-test,DESeq2,edgeR
hsa-miR-12136	4	NOISeq,Students t-test,DESeq2,edgeR
hsa-miR-141-3p	4	NOISeq,Students t-test,DESeq2,edgeR
hsa-miR-184	4	NOISeq,Students t-test,DESeq2,edgeR
hsa-miR-190a-5p	4	NOISeq,Students t-test,DESeq2,edgeR
hsa-miR-200a-3p	4	NOISeq,Students t-test,DESeq2,edgeR
hsa-miR-204-5p	4	NOISeq,Students t-test,DESeq2,edgeR
hsa-miR-30a-5p	4	DESeq,Students t-test,DESeq2,edgeR
hsa-miR-429	4	NOISeq,Students t-test,DESeq2,edgeR
hsa-miR-509-3-5p	4	NOISeq,Students t-test,DESeq2,edgeR
hsa-miR-514a-3p	4	NOISeq,Students t-test,DESeq2,edgeR
hsa-miR-101-3p	3	Students t-test,DESeq2,edgeR
hsa-miR-126-3p	3	Students t-test,DESeq2,edgeR
hsa-miR-126-5p	3	Students t-test,DESeq2,edgeR
hsa-miR-129-5p	3	Students t-test,DESeq2,edgeR
hsa-miR-138-5p	3	Students t-test,DESeq2,edgeR
hsa-miR-139-5p	3	Students t-test,DESeq2,edgeR
hsa-miR-140-5p	3	Students t-test,DESeq2,edgeR
hsa-miR-144-3p	3	Students t-test,DESeq2,edgeR
hsa-miR-144-5p	3	Students t-test,DESeq2,edgeR
hsa-miR-200a-5p	3	Students t-test,DESeq2,edgeR
hsa-miR-200b-3p	3	Students t-test,DESeq2,edgeR
hsa-miR-200c-3p	3	Students t-test,DESeq2,edgeR
hsa-miR-20b-5p	3	Students t-test,DESeq2,edgeR
hsa-miR-24-1-5p	3	Students t-test,DESeq2,edgeR
hsa-miR-27b-3p	3	Students t-test,DESeq2,edgeR
hsa-miR-29a-3p	3	Students t-test,DESeq2,edgeR
hsa-miR-29b-2-5p	3	Students t-test,DESeq2,edgeR
hsa-miR-29b-3p	3	Students t-test,DESeq2,edgeR
hsa-miR-29c-3p	3	Students t-test,DESeq2,edgeR
hsa-miR-29c-5p	3	Students t-test,DESeq2,edgeR
hsa-miR-30a-3p	3	Students t-test,DESeq2,edgeR
hsa-miR-30b-5p	3	Students t-test,DESeq2,edgeR
hsa-miR-30c-2-3p	3	Students t-test,DESeq2,edgeR
hsa-miR-30c-5p	3	Students t-test,DESeq2,edgeR
hsa-miR-30d-3p	3	Students t-test,DESeq2,edgeR
hsa-miR-30e-5p	3	Students t-test,DESeq2,edgeR
hsa-miR-338-3p	3	Students t-test,DESeq2,edgeR
hsa-miR-363-3p	3	Students t-test,DESeq2,edgeR
hsa-miR-378a-3p	3	Students t-test,DESeq2,edgeR
hsa-miR-378c	3	Students t-test,DESeq2,edgeR
hsa-miR-378d	3	Students t-test,DESeq2,edgeR
hsa-miR-378f	3	Students t-test,DESeq2,edgeR
hsa-miR-422a	3	Students t-test,DESeq2,edgeR
hsa-miR-455-5p	3	Students t-test,DESeq2,edgeR
hsa-miR-653-5p	3	Students t-test,DESeq2,edgeR
hsa-miR-9-5p	3	Students t-test,DESeq2,edgeR
hsa-miR-9985	3	Students t-test,DESeq2,edgeR

S4b) Increased expression of FFPE miRNA	*No. DE Tools*	*DE tools*
hsa-miR-1269a	5	DESeq,NOISeq,Students t-test,DESeq2,edgeR
hsa-miR-483-3p	5	DESeq,NOISeq,Students t-test,DESeq2,edgeR
hsa-miR-483-5p	5	DESeq,NOISeq,Students t-test,DESeq2,edgeR
hsa-miR-873-5p	5	DESeq,NOISeq,Students t-test,DESeq2,edgeR
hsa-miR-130b-5p	4	DESeq,Students t-test,DESeq2,edgeR
hsa-miR-323a-3p	4	DESeq,Students t-test,DESeq2,edgeR

hsa-let-7c-5p	3	Students t-test,DESeq2,edgeR
hsa-miR-1-3p	3	NOISeq,DESeq2,edgeR
hsa-miR-100-5p	3	Students t-test,DESeq2,edgeR
hsa-miR-106b-3p	3	Students t-test,DESeq2,edgeR
hsa-miR-125b-1-3p	3	Students t-test,DESeq2,edgeR
hsa-miR-125b-2-3p	3	Students t-test,DESeq2,edgeR
hsa-miR-125b-5p	3	Students t-test,DESeq2,edgeR
hsa-miR-127-3p	3	Students t-test,DESeq2,edgeR
hsa-miR-127-5p	3	Students t-test,DESeq2,edgeR
hsa-miR-1287-5p	3	Students t-test,DESeq2,edgeR
hsa-miR-130b-3p	3	NOISeq,DESeq2,edgeR
hsa-miR-132-3p	3	Students t-test,DESeq2,edgeR
hsa-miR-133a-3p	3	NOISeq,DESeq2,edgeR
hsa-miR-133b	3	NOISeq,DESeq2,edgeR
hsa-miR-134-5p	3	Students t-test,DESeq2,edgeR
hsa-miR-136-3p	3	Students t-test,DESeq2,edgeR
hsa-miR-152-3p	3	Students t-test,DESeq2,edgeR
hsa-miR-155-5p	3	Students t-test,DESeq2,edgeR
hsa-miR-181a-2-3p	3	Students t-test,DESeq2,edgeR
hsa-miR-181a-3p	3	Students t-test,DESeq2,edgeR
hsa-miR-181a-5p	3	Students t-test,DESeq2,edgeR
hsa-miR-181b-5p	3	Students t-test,DESeq2,edgeR
hsa-miR-181c-3p	3	Students t-test,DESeq2,edgeR
hsa-miR-181c-5p	3	Students t-test,DESeq2,edgeR
hsa-miR-181d-5p	3	Students t-test,DESeq2,edgeR
hsa-miR-196b-5p	3	Students t-test,DESeq2,edgeR
hsa-miR-199a-3p	3	Students t-test,DESeq2,edgeR
hsa-miR-199a-5p	3	Students t-test,DESeq2,edgeR
hsa-miR-199b-3p	3	Students t-test,DESeq2,edgeR
hsa-miR-199b-5p	3	Students t-test,DESeq2,edgeR
hsa-miR-206	3	NOISeq,DESeq2,edgeR
hsa-miR-214-3p	3	Students t-test,DESeq2,edgeR
hsa-miR-214-5p	3	Students t-test,DESeq2,edgeR
hsa-miR-218-5p	3	Students t-test,DESeq2,edgeR
hsa-miR-25-3p	3	Students t-test,DESeq2,edgeR
hsa-miR-331-5p	3	Students t-test,DESeq2,edgeR
hsa-miR-335-3p	3	Students t-test,DESeq2,edgeR
hsa-miR-335-5p	3	Students t-test,DESeq2,edgeR
hsa-miR-340-3p	3	Students t-test,DESeq2,edgeR
hsa-miR-340-5p	3	Students t-test,DESeq2,edgeR
hsa-miR-34a-5p	3	Students t-test,DESeq2,edgeR
hsa-miR-369-5p	3	Students t-test,DESeq2,edgeR
hsa-miR-370-3p	3	Students t-test,DESeq2,edgeR
hsa-miR-376c-3p	3	Students t-test,DESeq2,edgeR
hsa-miR-379-5p	3	Students t-test,DESeq2,edgeR
hsa-miR-381-3p	3	NOISeq,DESeq2,edgeR
hsa-miR-382-3p	3	Students t-test,DESeq2,edgeR
hsa-miR-382-5p	3	Students t-test,DESeq2,edgeR
hsa-miR-3913-5p	3	Students t-test,DESeq2,edgeR
hsa-miR-409-3p	3	Students t-test,DESeq2,edgeR
hsa-miR-409-5p	3	Students t-test,DESeq2,edgeR
hsa-miR-411-5p	3	Students t-test,DESeq2,edgeR
hsa-miR-421	3	Students t-test,DESeq2,edgeR
hsa-miR-432-5p	3	Students t-test,DESeq2,edgeR
hsa-miR-485-5p	3	Students t-test,DESeq2,edgeR
hsa-miR-493-3p	3	Students t-test,DESeq2,edgeR
hsa-miR-493-5p	3	Students t-test,DESeq2,edgeR
hsa-miR-494-3p	3	Students t-test,DESeq2,edgeR
hsa-miR-495-3p	3	Students t-test,DESeq2,edgeR
hsa-miR-503-5p	3	Students t-test,DESeq2,edgeR
hsa-miR-574-5p	3	Students t-test,DESeq2,edgeR
hsa-miR-654-3p	3	Students t-test,DESeq2,edgeR
hsa-miR-654-5p	3	Students t-test,DESeq2,edgeR
hsa-miR-671-5p	3	Students t-test,DESeq2,edgeR
hsa-miR-708-3p	3	Students t-test,DESeq2,edgeR
hsa-miR-708-5p	3	Students t-test,DESeq2,edgeR
hsa-miR-758-3p	3	Students t-test,DESeq2,edgeR
hsa-miR-887-3p	3	Students t-test,DESeq2,edgeR
hsa-miR-889-3p	3	Students t-test,DESeq2,edgeR
hsa-miR-99a-5p	3	Students t-test,DESeq2,edgeR

hsa-miR-99b-3p	3	Students t-test,DESeq2,edgeR

Supplementary Table S5: Differentially expressed miRNAs in FFT and FFPE HR, nHR and HK samples identified by at least three differential expression tools (sRNAde tool). Table 5Sa) shows decreased miRNA expression, and Table 4Sb) shows increased miRNA expression in compared sample groups (HR – High-Risk WT, nHR- non-High-Risk WT; HK – Healthy Kidney).

Table 5Sa) ***Decreased expression***

FFT HR vs nHR

name	*No. DE Tools*	*methods*
hsa-miR-133b	3	NOISeq,DESeq2,edgeR

FFT HR vs HK

name	*No. DE Tools*	*methods*
hsa-miR-141-3p	4	NOISeq,Students t-test,DESeq2,edgeR
hsa-miR-363-3p	3	Students t-test,DESeq2,edgeR
hsa-miR-139-5p	3	Students t-test,DESeq2,edgeR
hsa-miR-30a-3p	3	Students t-test,DESeq2,edgeR
hsa-miR-200a-3p	3	Students t-test,DESeq2,edgeR
hsa-miR-200a-5p	3	Students t-test,DESeq2,edgeR
hsa-miR-29c-3p	3	Students t-test,DESeq2,edgeR
hsa-miR-429	3	Students t-test,DESeq2,edgeR

FFPE HR vs nHR

name	*No. DE Tools*	*methods*
hsa-miR-379-5p	3	NOISeq,DESeq2,edgeR
hsa-miR-1269a	3	NOISeq,DESeq2,edgeR

FFPE HR vs HK

name	*No. DE Tools*	*methods*
hsa-miR-200c-3p	5	DESeq,NOISeq,Students t-test,DESeq2,edgeR
hsa-miR-141-3p	5	DESeq,NOISeq,Students t-test,DESeq2,edgeR
hsa-miR-509-3-5p	4	DESeq,NOISeq,DESeq2,edgeR
hsa-miR-139-5p	4	NOISeq,Students t-test,DESeq2,edgeR
hsa-miR-216b-5p	4	DESeq,NOISeq,DESeq2,edgeR
hsa-miR-934	4	DESeq,NOISeq,DESeq2,edgeR
hsa-miR-200a-5p	4	NOISeq,Students t-test,DESeq2,edgeR
hsa-miR-509-3p	4	DESeq,NOISeq,DESeq2,edgeR
hsa-miR-192-5p	4	DESeq,NOISeq,DESeq2,edgeR
hsa-miR-582-3p	4	NOISeq,Students t-test,DESeq2,edgeR
hsa-miR-514a-5p	4	DESeq,NOISeq,DESeq2,edgeR
hsa-miR-455-5p	4	NOISeq,Students t-test,DESeq2,edgeR
hsa-miR-217-5p	4	DESeq,NOISeq,DESeq2,edgeR
hsa-miR-509-5p	4	DESeq,NOISeq,DESeq2,edgeR
hsa-miR-141-5p	4	DESeq,Students t-test,DESeq2,edgeR
hsa-miR-429	4	NOISeq,Students t-test,DESeq2,edgeR
hsa-miR-514a-3p	4	DESeq,NOISeq,DESeq2,edgeR
hsa-miR-215-5p	4	DESeq,NOISeq,DESeq2,edgeR
hsa-miR-187-3p	4	DESeq,NOISeq,DESeq2,edgeR
hsa-miR-508-3p	4	DESeq,NOISeq,DESeq2,edgeR
hsa-miR-506-3p	3	NOISeq,DESeq2,edgeR
hsa-miR-455-3p	3	NOISeq,DESeq2,edgeR
hsa-miR-508-5p	3	NOISeq,DESeq2,edgeR
hsa-miR-5588-5p	3	Students t-test,DESeq2,edgeR
hsa-miR-532-3p	3	NOISeq,DESeq2,edgeR
hsa-miR-514b-3p	3	DESeq,DESeq2,edgeR
hsa-miR-1307-5p	3	NOISeq,DESeq2,edgeR
hsa-miR-139-3p	3	NOISeq,DESeq2,edgeR
hsa-miR-378a-3p	3	NOISeq,DESeq2,edgeR
hsa-miR-204-5p	3	NOISeq,DESeq2,edgeR
hsa-miR-200b-3p	3	NOISeq,Students t-test,DESeq2
hsa-miR-138-5p	3	NOISeq,DESeq2,edgeR
hsa-miR-200a-3p	3	NOISeq,Students t-test,edgeR
hsa-miR-362-5p	3	NOISeq,DESeq2,edgeR
hsa-miR-532-5p	3	NOISeq,DESeq2,edgeR
hsa-miR-194-5p	3	NOISeq,DESeq2,edgeR
hsa-miR-190a-5p	3	NOISeq,DESeq2,edgeR
hsa-miR-378c	3	NOISeq,DESeq2,edgeR
hsa-miR-378d	3	NOISeq,DESeq2,edgeR
hsa-miR-653-5p	3	NOISeq,DESeq2,edgeR
hsa-miR-184	3	NOISeq,DESeq2,edgeR
hsa-miR-12135	3	NOISeq,DESeq2,edgeR
hsa-miR-12136	3	NOISeq,DESeq2,edgeR
hsa-miR-194-3p	3	NOISeq,DESeq2,edgeR
hsa-miR-502-3p	3	NOISeq,DESeq2,edgeR
hsa-miR-363-3p	3	NOISeq,DESeq2,edgeR
hsa-miR-9-5p	3	NOISeq,DESeq2,edgeR
hsa-miR-513a-5p	3	DESeq,DESeq2,edgeR
hsa-miR-500a-3p	3	NOISeq,DESeq2,edgeR
hsa-miR-22-3p	3	NOISeq,DESeq2,edgeR
hsa-miR-514b-5p	3	DESeq,DESeq2,edgeR
hsa-miR-203a-3p	3	NOISeq,DESeq2,edgeR

Supplementary:
INTEGRATIVE TRANSCRIPTOMIC PROFILING OF WILMS TUMOR

FFT nHR vs HK

name	*No. DE Tools*	*methods*
hsa-miR-509-3-5p	5	DESeq,NOISeq,Students t-test,DESeq2,edgeR
hsa-miR-192-5p	5	DESeq,NOISeq,Students t-test,DESeq2,edgeR
hsa-miR-204-5p	5	DESeq,NOISeq,Students t-test,DESeq2,edgeR
hsa-miR-138-5p	5	DESeq,NOISeq,Students t-test,DESeq2,edgeR
hsa-miR-194-5p	5	DESeq,NOISeq,Students t-test,DESeq2,edgeR
hsa-miR-514a-3p	5	DESeq,NOISeq,Students t-test,DESeq2,edgeR
hsa-miR-12135	5	DESeq,NOISeq,Students t-test,DESeq2,edgeR
hsa-miR-215-5p	5	DESeq,NOISeq,Students t-test,DESeq2,edgeR
hsa-miR-187-3p	5	DESeq,NOISeq,Students t-test,DESeq2,edgeR
hsa-miR-508-3p	5	DESeq,NOISeq,Students t-test,DESeq2,edgeR
hsa-miR-30a-5p	5	DESeq,NOISeq,Students t-test,DESeq2,edgeR
hsa-miR-200a-3p	4	NOISeq,Students t-test,DESeq2,edgeR
hsa-miR-190a-5p	4	NOISeq,Students t-test,DESeq2,edgeR
hsa-miR-429	4	NOISeq,Students t-test,DESeq2,edgeR
hsa-miR-184	4	NOISeq,Students t-test,DESeq2,edgeR
hsa-miR-12136	4	NOISeq,Students t-test,DESeq2,edgeR
hsa-miR-29c-3p	4	NOISeq,Students t-test,DESeq2,edgeR
hsa-miR-139-5p	3	Students t-test,DESeq2,edgeR
hsa-miR-30c-5p	3	Students t-test,DESeq2,edgeR
hsa-miR-30a-3p	3	Students t-test,DESeq2,edgeR
hsa-miR-9985	3	Students t-test,DESeq2,edgeR
hsa-miR-200a-5p	3	Students t-test,DESeq2,edgeR
hsa-miR-30c-2-3p	3	Students t-test,DESeq2,edgeR
hsa-miR-1307-5p	3	Students t-test,DESeq2,edgeR
hsa-miR-29c-5p	3	Students t-test,DESeq2,edgeR
hsa-miR-378a-3p	3	Students t-test,DESeq2,edgeR
hsa-miR-30d-3p	3	Students t-test,DESeq2,edgeR
hsa-miR-29a-3p	3	Students t-test,DESeq2,edgeR
hsa-miR-200b-3p	3	Students t-test,DESeq2,edgeR
hsa-miR-6765-3p	3	Students t-test,DESeq2,edgeR
hsa-miR-422a	3	Students t-test,DESeq2,edgeR
hsa-miR-455-5p	3	Students t-test,DESeq2,edgeR
hsa-miR-30e-5p	3	Students t-test,DESeq2,edgeR
hsa-miR-144-5p	3	Students t-test,DESeq2,edgeR
hsa-miR-378c	3	Students t-test,DESeq2,edgeR
hsa-miR-378d	3	Students t-test,DESeq2,edgeR
hsa-miR-378e	3	Students t-test,DESeq2,edgeR
hsa-miR-653-5p	3	Students t-test,DESeq2,edgeR
hsa-miR-27b-3p	3	Students t-test,DESeq2,edgeR
hsa-miR-29a-5p	3	Students t-test,DESeq2,edgeR
hsa-miR-101-3p	3	Students t-test,DESeq2,edgeR
hsa-miR-378f	3	Students t-test,DESeq2,edgeR
hsa-miR-378g	3	Students t-test,DESeq2,edgeR
hsa-miR-378i	3	Students t-test,DESeq2,edgeR
hsa-miR-29b-3p	3	Students t-test,DESeq2,edgeR
hsa-miR-24-1-5p	3	Students t-test,DESeq2,edgeR
hsa-miR-200c-3p	3	Students t-test,DESeq2,edgeR
hsa-miR-144-3p	3	Students t-test,DESeq2,edgeR
hsa-miR-22-5p	3	Students t-test,DESeq2,edgeR
hsa-miR-30b-5p	3	Students t-test,DESeq2,edgeR
hsa-miR-141-3p	3	Students t-test,DESeq2,edgeR
hsa-miR-29b-2-5p	3	Students t-test,DESeq2,edgeR
hsa-miR-126-5p	3	Students t-test,DESeq2,edgeR
hsa-miR-363-3p	3	Students t-test,DESeq2,edgeR
hsa-miR-140-5p	3	Students t-test,DESeq2,edgeR
hsa-miR-338-3p	3	Students t-test,DESeq2,edgeR
hsa-miR-126-3p	3	Students t-test,DESeq2,edgeR

FFPE nHR vs HK

name	*No. DE Tools*	*methods*
hsa-miR-509-3-5p	5	DESeq,NOISeq,Students t-test,DESeq2,edgeR
hsa-miR-139-5p	5	DESeq,NOISeq,Students t-test,DESeq2,edgeR
hsa-miR-216b-5p	5	DESeq,NOISeq,Students t-test,DESeq2,edgeR
hsa-miR-509-3p	5	DESeq,NOISeq,Students t-test,DESeq2,edgeR
hsa-miR-30c-2-3p	5	DESeq,NOISeq,Students t-test,DESeq2,edgeR
hsa-miR-514b-3p	5	DESeq,NOISeq,Students t-test,DESeq2,edgeR
hsa-miR-192-5p	5	DESeq,NOISeq,Students t-test,DESeq2,edgeR
hsa-miR-204-5p	5	DESeq,NOISeq,Students t-test,DESeq2,edgeR
hsa-miR-514a-5p	5	DESeq,NOISeq,Students t-test,DESeq2,edgeR
hsa-miR-138-5p	5	DESeq,NOISeq,Students t-test,DESeq2,edgeR
hsa-miR-217-5p	5	DESeq,NOISeq,Students t-test,DESeq2,edgeR
hsa-miR-509-5p	5	DESeq,NOISeq,Students t-test,DESeq2,edgeR
hsa-miR-194-5p	5	DESeq,NOISeq,Students t-test,DESeq2,edgeR
hsa-miR-190a-5p	5	DESeq,NOISeq,Students t-test,DESeq2,edgeR
hsa-miR-514a-3p	5	DESeq,NOISeq,Students t-test,DESeq2,edgeR
hsa-miR-184	5	DESeq,NOISeq,Students t-test,DESeq2,edgeR
hsa-miR-12135	5	DESeq,NOISeq,Students t-test,DESeq2,edgeR
hsa-miR-29b-3p	5	DESeq,NOISeq,Students t-test,DESeq2,edgeR
hsa-miR-129-5p	5	DESeq,NOISeq,Students t-test,DESeq2,edgeR
hsa-miR-194-3p	5	DESeq,NOISeq,Students t-test,DESeq2,edgeR
hsa-miR-141-3p	5	DESeq,NOISeq,Students t-test,DESeq2,edgeR
hsa-miR-187-3p	5	DESeq,NOISeq,Students t-test,DESeq2,edgeR
hsa-miR-508-3p	5	DESeq,NOISeq,Students t-test,DESeq2,edgeR
hsa-miR-9-5p	5	DESeq,NOISeq,Students t-test,DESeq2,edgeR
hsa-miR-30a-5p	5	DESeq,NOISeq,Students t-test,DESeq2,edgeR
hsa-miR-203a-3p	5	DESeq,NOISeq,Students t-test,DESeq2,edgeR
hsa-miR-29c-3p	5	DESeq,NOISeq,Students t-test,DESeq2,edgeR
hsa-miR-506-3p	4	DESeq,NOISeq,DESeq2,edgeR
hsa-miR-892a	4	DESeq,Students t-test,DESeq2,edgeR
hsa-miR-508-5p	4	DESeq,NOISeq,DESeq2,edgeR
hsa-miR-30c-5p	4	NOISeq,Students t-test,DESeq2,edgeR
hsa-miR-30a-3p	4	NOISeq,Students t-test,DESeq2,edgeR
hsa-miR-934	4	DESeq,NOISeq,DESeq2,edgeR
hsa-miR-200a-5p	4	NOISeq,Students t-test,DESeq2,edgeR
hsa-miR-139-3p	4	NOISeq,Students t-test,DESeq2,edgeR
hsa-miR-378a-3p	4	NOISeq,Students t-test,DESeq2,edgeR
hsa-miR-29c-5p	4	NOISeq,Students t-test,DESeq2,edgeR
hsa-miR-29a-3p	4	NOISeq,Students t-test,DESeq2,edgeR
hsa-miR-200b-3p	4	NOISeq,Students t-test,DESeq2,edgeR
hsa-miR-200a-3p	4	DESeq,NOISeq,Students t-test,edgeR
hsa-miR-378c	4	NOISeq,Students t-test,DESeq2,edgeR
hsa-miR-141-5p	4	DESeq,Students t-test,DESeq2,edgeR
hsa-miR-429	4	NOISeq,Students t-test,DESeq2,edgeR
hsa-miR-510-5p	4	DESeq,Students t-test,DESeq2,edgeR
hsa-miR-200c-3p	4	NOISeq,Students t-test,DESeq2,edgeR
hsa-miR-22-5p	4	NOISeq,Students t-test,DESeq2,edgeR
hsa-miR-30b-5p	4	NOISeq,Students t-test,DESeq2,edgeR
hsa-miR-891a-5p	4	DESeq,Students t-test,DESeq2,edgeR
hsa-miR-22-3p	4	NOISeq,Students t-test,DESeq2,edgeR
hsa-miR-514b-5p	4	DESeq,Students t-test,DESeq2,edgeR
hsa-miR-216a-3p	4	DESeq,Students t-test,DESeq2,edgeR
hsa-miR-561-5p	4	DESeq,Students t-test,DESeq2,edgeR
hsa-miR-193a-3p	3	Students t-test,DESeq2,edgeR
hsa-miR-5588-5p	3	Students t-test,DESeq2,edgeR
hsa-miR-29b-1-5p	3	Students t-test,DESeq2,edgeR
hsa-miR-30d-3p	3	Students t-test,DESeq2,edgeR
hsa-miR-455-5p	3	Students t-test,DESeq2,edgeR
hsa-miR-422a	3	Students t-test,DESeq2,edgeR
hsa-miR-362-5p	3	NOISeq,DESeq2,edgeR
hsa-miR-888-5p	3	Students t-test,DESeq2,edgeR
hsa-miR-532-5p	3	Students t-test,DESeq2,edgeR
hsa-miR-378d	3	NOISeq,DESeq2,edgeR
hsa-miR-192-3p	3	DESeq,DESeq2,edgeR
hsa-miR-378e	3	Students t-test,DESeq2,edgeR
hsa-miR-378a-5p	3	Students t-test,DESeq2,edgeR
hsa-miR-101-3p	3	Students t-test,DESeq2,edgeR
hsa-miR-12136	3	NOISeq,DESeq2,edgeR
hsa-miR-378f	3	Students t-test,DESeq2,edgeR
hsa-miR-378g	3	Students t-test,DESeq2,edgeR
hsa-miR-378h	3	Students t-test,DESeq2,edgeR
hsa-miR-378i	3	Students t-test,DESeq2,edgeR
hsa-miR-6500-3p	3	Students t-test,DESeq2,edgeR
hsa-miR-215-5p	3	NOISeq,DESeq2,edgeR
hsa-miR-216a-5p	3	Students t-test,DESeq2,edgeR

hsa-miR-126-5p	3	Students t-test,DESeq2,edgeR
hsa-miR-23a-5p	3	Students t-test,DESeq2,edgeR
hsa-miR-513a-5p	3	DESeq,DESeq2,edgeR
hsa-miR-30d-5p	3	Students t-test,DESeq2,edgeR
hsa-miR-200b-5p	3	Students t-test,DESeq2,edgeR
hsa-miR-126-3p	3	Students t-test,DESeq2,edgeR

Table 5Sb) ***Increased expression***

FFT HR vs nHR

name	*No. DE Tools*	*methods*
hsa-miR-892a	3	DESeq,DESeq2,edgeR

FFPE HR vs nHR

name	*No. DE Tools*	*methods*
hsa-miR-514a-3p	4	DESeq,NOISeq,DESeq2,edgeR
hsa-miR-508-3p	4	DESeq,NOISeq,DESeq2,edgeR
hsa-miR-192-5p	3	DESeq,NOISeq,edgeR
hsa-miR-509-3-5p	3	DESeq,NOISeq,edgeR

FFT HR vs HK

name	*No. DE Tools*	*methods*
hsa-miR-93-5p	4	DESeq,NOISeq,DESeq2,edgeR
hsa-miR-301b-3p	4	DESeq,NOISeq,DESeq2,edgeR
hsa-miR-3085-3p	4	DESeq,Students t-test,DESeq2,edgeR
hsa-miR-516a-5p	4	DESeq,Students t-test,DESeq2,edgeR
hsa-miR-135a-3p	4	DESeq,NOISeq,DESeq2,edgeR
hsa-miR-149-5p	4	DESeq,NOISeq,DESeq2,edgeR
hsa-miR-1238-3p	4	DESeq,Students t-test,DESeq2,edgeR
hsa-miR-106b-3p	4	DESeq,NOISeq,DESeq2,edgeR
hsa-miR-6788-3p	3	Students t-test,DESeq2,edgeR
hsa-miR-6868-3p	3	NOISeq,DESeq2,edgeR
hsa-miR-181a-2-3p	3	Students t-test,DESeq2,edgeR
hsa-miR-20a-3p	3	Students t-test,DESeq2,edgeR
hsa-miR-6759-5p	3	Students t-test,DESeq2,edgeR
hsa-miR-17-5p	3	NOISeq,DESeq2,edgeR
hsa-miR-196a-3p	3	NOISeq,DESeq2,edgeR
hsa-miR-183-5p	3	NOISeq,DESeq2,edgeR
hsa-miR-877-5p	3	NOISeq,DESeq2,edgeR
hsa-miR-4498	3	DESeq,DESeq2,edgeR
hsa-miR-483-3p	3	NOISeq,DESeq2,edgeR
hsa-miR-196a-5p	3	NOISeq,DESeq2,edgeR
hsa-miR-135a-5p	3	NOISeq,DESeq2,edgeR
hsa-miR-873-3p	3	DESeq,DESeq2,edgeR
hsa-miR-96-5p	3	NOISeq,DESeq2,edgeR
hsa-miR-128-3p	3	NOISeq,DESeq2,edgeR
hsa-miR-6818-5p	3	DESeq,DESeq2,edgeR
hsa-miR-490-3p	3	DESeq,DESeq2,edgeR
hsa-miR-18a-5p	3	DESeq,DESeq2,edgeR
hsa-miR-1197	3	DESeq,DESeq2,edgeR
hsa-miR-873-5p	3	DESeq,DESeq2,edgeR
hsa-miR-3059-5p	3	DESeq,DESeq2,edgeR
hsa-miR-483-5p	3	NOISeq,DESeq2,edgeR
hsa-miR-153-3p	3	Students t-test,DESeq2,edgeR
hsa-miR-342-3p	3	NOISeq,DESeq2,edgeR
hsa-miR-6516-5p	3	NOISeq,DESeq2,edgeR
hsa-miR-708-3p	3	Students t-test,DESeq2,edgeR
hsa-miR-182-3p	3	NOISeq,DESeq2,edgeR
hsa-miR-206	3	NOISeq,DESeq2,edgeR
hsa-miR-323b-3p	3	NOISeq,DESeq2,edgeR
hsa-miR-424-3p	3	NOISeq,DESeq2,edgeR

FFPE HR vs HK

name	*No. DE Tools*	*methods*
hsa-miR-483-3p	5	DESeq,NOISeq,Students t-test,DESeq2,edgeR
hsa-miR-483-5p	5	DESeq,NOISeq,Students t-test,DESeq2,edgeR
hsa-miR-130b-3p	4	NOISeq,Students t-test,DESeq2,edgeR
hsa-miR-873-5p	4	NOISeq,Students t-test,DESeq2,edgeR
hsa-miR-1269a	4	NOISeq,Students t-test,DESeq2,edgeR
hsa-miR-199b-3p	3	Students t-test,DESeq2,edgeR
hsa-miR-214-3p	3	Students t-test,DESeq2,edgeR
hsa-miR-301a-3p	3	Students t-test,DESeq2,edgeR
hsa-miR-671-5p	3	Students t-test,DESeq2,edgeR
hsa-miR-147b-3p	3	Students t-test,DESeq2,edgeR
hsa-miR-4677-3p	3	Students t-test,DESeq2,edgeR
hsa-miR-708-3p	3	Students t-test,DESeq2,edgeR
hsa-miR-196b-5p	3	Students t-test,DESeq2,edgeR
hsa-miR-370-3p	3	Students t-test,DESeq2,edgeR
hsa-miR-199a-3p	3	Students t-test,DESeq2,edgeR
hsa-miR-106b-5p	3	Students t-test,DESeq2,edgeR
hsa-miR-382-5p	3	Students t-test,DESeq2,edgeR
hsa-miR-493-3p	3	Students t-test,DESeq2,edgeR
hsa-miR-218-5p	3	Students t-test,DESeq2,edgeR
hsa-miR-1278	3	Students t-test,DESeq2,edgeR
hsa-miR-214-5p	3	Students t-test,DESeq2,edgeR
hsa-miR-181b-5p	3	Students t-test,DESeq2,edgeR
hsa-miR-548ay-5p	3	Students t-test,DESeq2,edgeR
hsa-miR-130b-5p	3	Students t-test,DESeq2,edgeR
hsa-miR-432-5p	3	Students t-test,DESeq2,edgeR
hsa-miR-125b-1-3p	3	Students t-test,DESeq2,edgeR
hsa-miR-632	3	Students t-test,DESeq2,edgeR
hsa-miR-628-5p	3	Students t-test,DESeq2,edgeR
hsa-miR-708-5p	3	Students t-test,DESeq2,edgeR
hsa-miR-758-3p	3	Students t-test,DESeq2,edgeR
hsa-miR-135b-3p	3	Students t-test,DESeq2,edgeR
hsa-miR-18a-3p	3	Students t-test,DESeq2,edgeR
hsa-miR-181a-3p	3	Students t-test,DESeq2,edgeR
hsa-miR-199a-5p	3	Students t-test,DESeq2,edgeR
hsa-miR-3912-3p	3	Students t-test,DESeq2,edgeR
hsa-miR-665	3	Students t-test,DESeq2,edgeR

hsa-miR-199b-5p	3	NOISeq,DESeq2,edgeR
hsa-miR-7706	3	NOISeq,DESeq2,edgeR
hsa-miR-130b-5p	3	NOISeq,DESeq2,edgeR
hsa-miR-490-5p	3	DESeq,DESeq2,edgeR
hsa-miR-182-5p	3	NOISeq,DESeq2,edgeR
hsa-miR-1843	3	NOISeq,DESeq2,edgeR
hsa-miR-4673	3	Students t-test,DESeq2,edgeR
hsa-miR-3118	3	DESeq,DESeq2,edgeR
hsa-miR-1226-3p	3	NOISeq,DESeq2,edgeR

FFT nHR vs HK

name	*No. DE Tools*	*methods*
hsa-miR-135a-3p	5	DESeq,NOISeq,Students t-test,DESeq2,edgeR
hsa-miR-490-3p	5	DESeq,NOISeq,Students t-test,DESeq2,edgeR
hsa-miR-873-5p	5	DESeq,NOISeq,Students t-test,DESeq2,edgeR
hsa-miR-323a-3p	5	DESeq,NOISeq,Students t-test,DESeq2,edgeR
hsa-miR-199b-5p	5	DESeq,NOISeq,Students t-test,DESeq2,edgeR
hsa-miR-335-3p	5	DESeq,NOISeq,Students t-test,DESeq2,edgeR
hsa-miR-876-3p	4	DESeq,Students t-test,DESeq2,edgeR
hsa-miR-301b-3p	4	DESeq,NOISeq,DESeq2,edgeR
hsa-miR-181b-3p	4	DESeq,Students t-test,DESeq2,edgeR
hsa-miR-1269a	4	DESeq,NOISeq,DESeq2,edgeR
hsa-miR-873-3p	4	DESeq,NOISeq,DESeq2,edgeR
hsa-miR-6818-5p	4	DESeq,Students t-test,DESeq2,edgeR
hsa-miR-18a-5p	4	DESeq,Students t-test,DESeq2,edgeR
hsa-miR-1197	4	DESeq,Students t-test,DESeq2,edgeR
hsa-miR-130b-3p	4	DESeq,NOISeq,DESeq2,edgeR
hsa-miR-1276	4	DESeq,Students t-test,DESeq2,edgeR
hsa-miR-130b-5p	4	DESeq,NOISeq,DESeq2,edgeR
hsa-miR-490-5p	4	DESeq,Students t-test,DESeq2,edgeR
hsa-miR-548l	3	Students t-test,DESeq2,edgeR
hsa-miR-3622a-5p	3	Students t-test,DESeq2,edgeR
hsa-miR-744-3p	3	Students t-test,DESeq2,edgeR
hsa-miR-6735-5p	3	Students t-test,DESeq2,edgeR
hsa-miR-876-5p	3	Students t-test,DESeq2,edgeR
hsa-miR-376c-5p	3	Students t-test,DESeq2,edgeR
hsa-miR-493-3p	3	Students t-test,DESeq2,edgeR
hsa-miR-181a-2-3p	3	Students t-test,DESeq2,edgeR
hsa-miR-301a-5p	3	Students t-test,DESeq2,edgeR
hsa-miR-1304-5p	3	Students t-test,DESeq2,edgeR
hsa-miR-17-5p	3	Students t-test,DESeq2,edgeR
hsa-miR-181a-3p	3	NOISeq,DESeq2,edgeR
hsa-miR-3912-3p	3	Students t-test,DESeq2,edgeR
hsa-miR-4659a-3p	3	Students t-test,DESeq2,edgeR
hsa-miR-6505-5p	3	Students t-test,DESeq2,edgeR
hsa-miR-183-5p	3	NOISeq,DESeq2,edgeR
hsa-miR-1247-3p	3	Students t-test,DESeq2,edgeR
hsa-miR-877-5p	3	Students t-test,DESeq2,edgeR
hsa-let-7a-2-3p	3	Students t-test,DESeq2,edgeR
hsa-miR-597-3p	3	Students t-test,DESeq2,edgeR
hsa-miR-494-3p	3	Students t-test,DESeq2,edgeR
hsa-miR-4498	3	DESeq,DESeq2,edgeR
hsa-miR-1302	3	Students t-test,DESeq2,edgeR
hsa-miR-3064-5p	3	Students t-test,DESeq2,edgeR
hsa-miR-199a-3p	3	Students t-test,DESeq2,edgeR
hsa-miR-431-3p	3	NOISeq,DESeq2,edgeR
hsa-miR-1185-1-3p	3	Students t-test,DESeq2,edgeR
hsa-miR-382-5p	3	Students t-test,DESeq2,edgeR
hsa-miR-483-3p	3	NOISeq,DESeq2,edgeR
hsa-miR-196a-5p	3	NOISeq,DESeq2,edgeR
hsa-miR-3174	3	Students t-test,DESeq2,edgeR

FFPE nHR vs HK

name	*No. DE Tools*	*methods*
hsa-miR-873-5p	5	DESeq,NOISeq,Students t-test,DESeq2,edgeR
hsa-miR-483-5p	5	DESeq,NOISeq,Students t-test,DESeq2,edgeR
hsa-miR-483-3p	5	DESeq,NOISeq,Students t-test,DESeq2,edgeR
hsa-miR-323a-3p	5	DESeq,NOISeq,Students t-test,DESeq2,edgeR
hsa-miR-1269a	5	DESeq,NOISeq,Students t-test,DESeq2,edgeR
hsa-miR-433-3p	4	DESeq,Students t-test,DESeq2,edgeR
hsa-miR-199b-5p	4	NOISeq,Students t-test,DESeq2,edgeR
hsa-miR-199b-3p	3	Students t-test,DESeq2,edgeR
hsa-miR-3913-5p	3	Students t-test,DESeq2,edgeR
hsa-miR-340-5p	3	Students t-test,DESeq2,edgeR
hsa-miR-214-3p	3	Students t-test,DESeq2,edgeR
hsa-miR-133b	3	NOISeq,DESeq2,edgeR
hsa-miR-331-5p	3	Students t-test,DESeq2,edgeR
hsa-miR-337-3p	3	Students t-test,DESeq2,edgeR
hsa-miR-381-3p	3	NOISeq,DESeq2,edgeR
hsa-miR-196b-5p	3	Students t-test,DESeq2,edgeR
hsa-miR-127-5p	3	Students t-test,DESeq2,edgeR
hsa-miR-370-3p	3	Students t-test,DESeq2,edgeR
hsa-miR-152-3p	3	Students t-test,DESeq2,edgeR
hsa-miR-181c-3p	3	Students t-test,DESeq2,edgeR
hsa-miR-99a-5p	3	Students t-test,DESeq2,edgeR
hsa-miR-503-5p	3	Students t-test,DESeq2,edgeR
hsa-let-7c-5p	3	Students t-test,DESeq2,edgeR
hsa-miR-125b-2-3p	3	Students t-test,DESeq2,edgeR
hsa-miR-154-5p	3	Students t-test,DESeq2,edgeR
hsa-miR-125b-5p	3	Students t-test,DESeq2,edgeR
hsa-miR-329-3p	3	Students t-test,DESeq2,edgeR
hsa-miR-493-3p	3	Students t-test,DESeq2,edgeR
hsa-miR-181a-2-3p	3	Students t-test,DESeq2,edgeR
hsa-miR-369-5p	3	Students t-test,DESeq2,edgeR
hsa-miR-214-5p	3	Students t-test,DESeq2,edgeR
hsa-miR-487b-3p	3	Students t-test,DESeq2,edgeR
hsa-miR-495-3p	3	Students t-test,DESeq2,edgeR
hsa-miR-432-5p	3	Students t-test,DESeq2,edgeR
hsa-miR-136-3p	3	Students t-test,DESeq2,edgeR
hsa-miR-758-3p	3	Students t-test,DESeq2,edgeR
hsa-miR-93-3p	3	Students t-test,DESeq2,edgeR
hsa-miR-574-5p	3	Students t-test,DESeq2,edgeR
hsa-miR-299-5p	3	Students t-test,DESeq2,edgeR
hsa-miR-4510	3	Students t-test,DESeq2,edgeR
hsa-miR-181a-3p	3	Students t-test,DESeq2,edgeR
hsa-miR-127-3p	3	Students t-test,DESeq2,edgeR
hsa-miR-199a-5p	3	Students t-test,DESeq2,edgeR
hsa-miR-181c-5p	3	Students t-test,DESeq2,edgeR
hsa-miR-3912-3p	3	Students t-test,DESeq2,edgeR
hsa-miR-769-5p	3	Students t-test,DESeq2,edgeR
hsa-miR-665	3	Students t-test,DESeq2,edgeR
hsa-miR-382-3p	3	Students t-test,DESeq2,edgeR
hsa-miR-181d-5p	3	Students t-test,DESeq2,edgeR

hsa-miR-409-5p	3	Students t-test,DESeq2,edgeR
hsa-miR-181b-5p	3	NOISeq,DESeq2,edgeR
hsa-miR-6877-5p	3	Students t-test,DESeq2,edgeR
hsa-miR-15b-3p	3	Students t-test,DESeq2,edgeR
hsa-miR-205-5p	3	Students t-test,DESeq2,edgeR
hsa-miR-431-5p	3	NOISeq,DESeq2,edgeR
hsa-miR-4286	3	Students t-test,DESeq2,edgeR
hsa-miR-96-5p	3	NOISeq,DESeq2,edgeR
hsa-miR-199b-3p	3	Students t-test,DESeq2,edgeR
hsa-miR-1185-2-3p	3	Students t-test,DESeq2,edgeR
hsa-miR-196b-5p	3	Students t-test,DESeq2,edgeR
hsa-miR-454-5p	3	Students t-test,DESeq2,edgeR
hsa-miR-376a-3p	3	Students t-test,DESeq2,edgeR
hsa-miR-6807-5p	3	Students t-test,DESeq2,edgeR
hsa-miR-329-3p	3	Students t-test,DESeq2,edgeR
hsa-miR-1228-3p	3	Students t-test,DESeq2,edgeR
hsa-miR-6721-5p	3	Students t-test,DESeq2,edgeR
hsa-miR-6514-5p	3	Students t-test,DESeq2,edgeR
hsa-miR-4470	3	Students t-test,DESeq2,edgeR
hsa-miR-758-3p	3	Students t-test,DESeq2,edgeR
hsa-miR-127-3p	3	Students t-test,DESeq2,edgeR
hsa-miR-370-5p	3	Students t-test,DESeq2,edgeR
hsa-miR-548ba	3	NOISeq,DESeq2,edgeR
hsa-miR-433-3p	3	NOISeq,DESeq2,edgeR
hsa-miR-483-5p	3	NOISeq,DESeq2,edgeR
hsa-miR-382-3p	3	Students t-test,DESeq2,edgeR
hsa-miR-5695	3	Students t-test,DESeq2,edgeR
hsa-miR-301a-3p	3	Students t-test,DESeq2,edgeR
hsa-miR-6516-5p	3	Students t-test,DESeq2,edgeR
hsa-miR-654-3p	3	Students t-test,DESeq2,edgeR
hsa-miR-335-5p	3	Students t-test,DESeq2,edgeR
hsa-miR-3177-3p	3	Students t-test,DESeq2,edgeR
hsa-miR-412-5p	3	DESeq,DESeq2,edgeR
hsa-miR-206	3	NOISeq,DESeq2,edgeR
hsa-miR-7706	3	Students t-test,DESeq2,edgeR
hsa-miR-125b-1-3p	3	Students t-test,DESeq2,edgeR
hsa-miR-654-5p	3	Students t-test,DESeq2,edgeR
hsa-miR-708-5p	3	Students t-test,DESeq2,edgeR
hsa-miR-182-5p	3	NOISeq,DESeq2,edgeR
hsa-miR-6764-5p	3	Students t-test,DESeq2,edgeR
hsa-miR-3115	3	DESeq,DESeq2,edgeR
hsa-miR-3118	3	DESeq,DESeq2,edgeR
hsa-miR-181b-2-3p	3	Students t-test,DESeq2,edgeR
hsa-miR-380-5p	3	Students t-test,DESeq2,edgeR
hsa-miR-18b-5p	3	Students t-test,DESeq2,edgeR
hsa-miR-106b-3p	3	Students t-test,DESeq2,edgeR
hsa-miR-1226-3p	3	Students t-test,DESeq2,edgeR
hsa-miR-4443	3	Students t-test,DESeq2,edgeR
hsa-miR-16-2-3p	3	Students t-test,DESeq2,edgeR

hsa-miR-301a-3p	3	Students t-test,DESeq2,edgeR
hsa-miR-671-5p	3	Students t-test,DESeq2,edgeR
hsa-miR-1287-5p	3	Students t-test,DESeq2,edgeR
hsa-miR-654-3p	3	Students t-test,DESeq2,edgeR
hsa-miR-335-5p	3	Students t-test,DESeq2,edgeR
hsa-miR-494-3p	3	Students t-test,DESeq2,edgeR
hsa-miR-708-3p	3	Students t-test,DESeq2,edgeR
hsa-miR-1296-5p	3	Students t-test,DESeq2,edgeR
hsa-miR-199a-3p	3	Students t-test,DESeq2,edgeR
hsa-miR-450a-2-3p	3	Students t-test,DESeq2,edgeR
hsa-miR-181a-5p	3	Students t-test,DESeq2,edgeR
hsa-miR-1-3p	3	NOISeq,DESeq2,edgeR
hsa-miR-410-3p	3	NOISeq,DESeq2,edgeR
hsa-miR-382-5p	3	Students t-test,DESeq2,edgeR
hsa-miR-485-5p	3	Students t-test,DESeq2,edgeR
hsa-miR-889-3p	3	Students t-test,DESeq2,edgeR
hsa-miR-218-5p	3	Students t-test,DESeq2,edgeR
hsa-miR-409-5p	3	Students t-test,DESeq2,edgeR
hsa-miR-424-3p	3	Students t-test,DESeq2,edgeR
hsa-miR-25-3p	3	Students t-test,DESeq2,edgeR
hsa-miR-132-3p	3	Students t-test,DESeq2,edgeR
hsa-miR-181b-5p	3	Students t-test,DESeq2,edgeR
hsa-miR-887-3p	3	Students t-test,DESeq2,edgeR
hsa-miR-134-5p	3	Students t-test,DESeq2,edgeR
hsa-miR-130b-5p	3	Students t-test,DESeq2,edgeR
hsa-miR-125b-1-3p	3	Students t-test,DESeq2,edgeR
hsa-miR-654-5p	3	Students t-test,DESeq2,edgeR
hsa-miR-379-5p	3	Students t-test,DESeq2,edgeR
hsa-miR-335-3p	3	Students t-test,DESeq2,edgeR
hsa-miR-708-5p	3	Students t-test,DESeq2,edgeR
hsa-miR-1271-5p	3	Students t-test,DESeq2,edgeR
hsa-miR-100-5p	3	Students t-test,DESeq2,edgeR
hsa-miR-376c-3p	3	Students t-test,DESeq2,edgeR
hsa-miR-431-5p	3	NOISeq,DESeq2,edgeR
hsa-miR-34a-5p	3	Students t-test,DESeq2,edgeR
hsa-miR-493-5p	3	Students t-test,DESeq2,edgeR
hsa-miR-409-3p	3	Students t-test,DESeq2,edgeR
hsa-miR-4324	3	Students t-test,DESeq2,edgeR

Supplementary:
INTEGRATIVE TRANSCRIPTOMIC PROFILING OF WILMS TUMOR

Supplementary Table S6: *Cross-sectional analysis of FFT and FFPE differential expression* miRNAs of HR, nHR and HR WT samples. We did not identify significant miRNAs for HR WT when HR samples were compared to nHR. In contrast, by comparing HR WT and nHR WT with HK samples, we identified 5 miRNAs with de-creased expression and 6 miRNAs with increased expression in HR WT (HR – High-Risk WT, nHR- non-High-Risk WT; HK – Healthy Kidney).

Decreased expression in WT tissue	*Increased expression in WT tissue*
HR vs nHR	*HR vs nHR*
/	/
HR vs HK	*HR vs HK*
hsa-miR-483-3p	hsa-miR-141-3p
hsa-miR-483-5p	hsa-miR-363-3p
hsa-miR-873-5p	hsa-miR-139-5p
hsa-miR-708-3p	hsa-miR-200a-3p
hsa-miR-130b-5p	hsa-miR-200a-5p
	hsa-miR-429
nHR vs HK	*nHR vs HK*
hsa-miR-873-5p	hsa-miR-509-3-5p
hsa-miR-483-5p	hsa-miR-192-5p
hsa-miR-483-3p	hsa-miR-204-5p
hsa-miR-323a-3p	hsa-miR-138-5p
hsa-miR-1269a	hsa-miR-194-5p
hsa-miR-433-3p	hsa-miR-514a-3p
hsa-miR-199b-5p	hsa-miR-12135
hsa-miR-199b-3p	hsa-miR-215-5p
hsa-miR-196b-5p	hsa-miR-187-3p
hsa-miR-329-3p	hsa-miR-508-3p
hsa-miR-493-3p	hsa-miR-30a-5p
hsa-miR-181a-2-3p	hsa-miR-200a-3p
hsa-miR-758-3p	hsa-miR-190a-5p
hsa-miR-181a-3p	hsa-miR-429
hsa-miR-127-3p	hsa-miR-184
hsa-miR-3912-3p	hsa-miR-12136
hsa-miR-382-3p	hsa-miR-29c-3p
hsa-miR-301a-3p	hsa-miR-139-5p
hsa-miR-654-3p	hsa-miR-30c-5p
hsa-miR-335-5p	hsa-miR-30a-3p
hsa-miR-494-3p	hsa-miR-200a-5p
hsa-miR-199a-3p	hsa-miR-30c-2-3p
hsa-miR-382-5p	hsa-miR-29c-5p
hsa-miR-409-5p	hsa-miR-378a-3p
hsa-miR-181b-5p	hsa-miR-30d-3p
hsa-miR-130b-5p	hsa-miR-29a-3p
hsa-miR-125b-1-3p	hsa-miR-200b-3p
hsa-miR-654-5p	hsa-miR-422a
hsa-miR-335-3p	hsa-miR-455-5p
hsa-miR-708-5p	hsa-miR-378c
hsa-miR-431-5p	hsa-miR-378d
	hsa-miR-378e
	hsa-miR-101-3p
	hsa-miR-378f
	hsa-miR-378g
	hsa-miR-378i
	hsa-miR-29b-3p
	hsa-miR-200c-3p
	hsa-miR-22-5p
	hsa-miR-30b-5p
	hsa-miR-141-3p
	hsa-miR-126-5p
	hsa-miR-126-3p

Supplementary:
INTEGRATIVE TRANSCRIPTOMIC PROFILING OF WILMS TUMOR

Supplementary Table S8: Predicted mRNAs regulated by differentially expressed miRNAs identified by miRnet in silico minimal network pathway analysis. Highlighted mRNA transcripts in red represent differentially expressed transcripts in the WT, as confirmed by mRNA sequencing analysis.

**Prostate cancer**	**Focal adhesion**	**Pathways in cancer**	**Bladder cancer**	**Glioma**	**p53 signaling pathway**	**Melanoma**	**Chronic myeloid leukemia**	**Small cell lung cancer**	**Apoptosis**	**Epstein-Barr virus infection**
CCND1	*CCND1*	*FAS*	*CCND1*	*CCND1*	*FAS*	*CCND1*	*CCND1*	*CCND1*	*FAS*	*RBPJ*
BCL2	*BCL2*	*CCND1*	*ERBB2*	*AKT1*	*ATM*	*AKT1*	*AKT1*	*AKT1*	*ATM*	*AKT1*
AKT1	*DIAPH1*	*BCL2*	*MDM2*	*IGF1R*	*CCND1*	*IGF1R*	*CDKN1B*	*CDKN1B*	*BCL2*	*CDKN1B*
CDKN1B	*AKT1*	*HIF1A*		*MDM2*	*MDM2*	*MDM2*	*MDM2*	*ITGA3*	*AKT1*	*MDM2*
IGF1R	*CAV1*	*AKT1*								
ERBB2	*IGF1R*	*CDKN1B*								
MDM2	*ITGA3*	*IGF1R*								
	ERBB2	*ITGA3*								
	VAV3	*ERBB2*								
		MDM2								
